# AI-Enabled Piezoelectric Wearable for Joint Torque Monitoring

**DOI:** 10.1007/s40820-025-01753-w

**Published:** 2025-05-03

**Authors:** Jinke Chang, Jinchen Li, Jiahao Ye, Bowen Zhang, Jianan Chen, Yunjia Xia, Jingyu Lei, Tom Carlson, Rui Loureiro, Alexander M. Korsunsky, Jin-Chong Tan, Hubin Zhao

**Affiliations:** 1 Multifunctional Materials and Composites (MMC) Laboratory, Department of Engineering Science, University of Oxford, Oxford, OX1 3PJ UK; 2 HUB of Intelligent Neuro-Engineering (HUBIN), Aspire CREATe, DSIS, University College London, London, HA7 4LP UK; 3 State Key Laboratory for Manufacturing System Engineering, School of Mechanical Engineering, Xi’an Jiaotong University, Xi’an, 710054 People’s Republic of China; 4 Trinity College, University of Oxford, Oxford, OX1 3BH UK

**Keywords:** Artificial intelligence wearables, Joint torque monitoring, Boron nitride nanotubes, Piezoelectric devices, Inverse design

## Abstract

Joint health is critical for musculoskeletal (MSK) conditions that are affecting approximately one-third of the global population. Monitoring of joint torque can offer an important pathway for the evaluation of joint health and guided intervention. However, there is no technology that can provide the precision, effectiveness, low-resource setting, and long-term wearability to simultaneously achieve both rapid and accurate joint torque measurement to enable risk assessment of joint injury and long-term monitoring of joint rehabilitation in wider environments. Herein, we propose a piezoelectric boron nitride nanotubes (BNNTs)-based, AI-enabled wearable device for regular monitoring of joint torque. We first adopted an iterative inverse design to fabricate the wearable materials with a Poisson’s ratio precisely matched to knee biomechanics. A highly sensitive piezoelectric film was constructed based on BNNTs and polydimethylsiloxane and applied to precisely capture the knee motion, while concurrently realizing self-sufficient energy harvesting. With the help of a lightweight on-device artificial neural network, the proposed wearable device was capable of accurately extracting targeted signals from the complex piezoelectric outputs and then effectively mapping these signals to their corresponding physical characteristics, including torque, angle, and loading. A real-time platform was constructed to demonstrate the capability of fine real-time torque estimation. This work offers a relatively low-cost wearable solution for effective, regular joint torque monitoring that can be made accessible to diverse populations in countries and regions with heterogeneous development levels, potentially producing wide-reaching global implications for joint health, MSK conditions, ageing, rehabilitation, personal health, and beyond.

**Supplementary Information:**

The online version contains supplementary material available at 10.1007/s40820-025-01753-w.

## Highlights

- Artificial intelligence-enabled wearable device with boron nitride nanotubes (BNNTs)-based piezoelectric film for accurate joint torque sensing.
- Inverse-designed structure optimizes biomechanical compatibility for enhanced knee motion tracking.
- High-sensitivity BNNTs/polydimethylsiloxane composite enables precise and dynamic knee motion signal detection.
- Lightweight neural network processes complex signals for accurate torque, angle, and load estimation.
- Real-time monitoring system provides instant knee torque assessment for daily use.

**Supplementary Information:**

The online version contains supplementary material available at 10.1007/s40820-025-01753-w.

## Graphical abstract

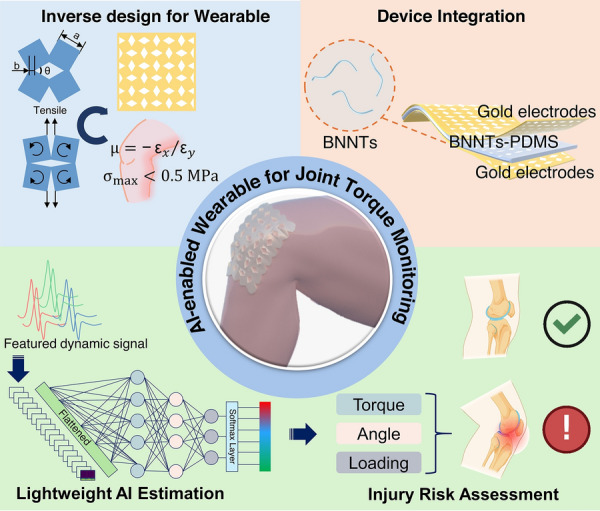

## Introduction

Musculoskeletal (MSK) conditions are a leading cause of disability worldwide, affecting approximately 1.71 billion people in 2019 and placing a significant burden on healthcare systems and economies [1–4]. Among the major MSK disorders, joint-related conditions such as osteoarthritis and rheumatoid arthritis significantly impact mobility and rehabilitation needs [5, 6]. These conditions not only compromise joint stability but also increase susceptibility to injuries, creating a cycle that leads to chronic pain, reduced function, and long-term disability [7]. Given the increasing prevalence of MSK disorders, particularly among high-risk populations such as the elderly and individuals with obesity, reliable methods for joint health monitoring are essential [2, 8–11].

A key factor in joint health assessment is the ability to quantify joint torque, which plays a crucial role in understanding internal joint mechanics, injury risk, and rehabilitation progress [12, 13]. Joint torque, influenced by joint angles, motion speed, external loads, and muscle activation, directly reflects internal joint stresses [13]. Excessive torque, particularly in joints like the knee, is a primary factor in injuries such as ligament tears, meniscus damage, and tendon overload [14, 15]. The knee joint, classified as a modified hinge joint, is one of the most mechanically complex load-bearing structures in the human body [16]. It primarily provides two degrees of freedom: flexion–extension and axial rotation. Flexion and extension are the dominant motions, typically ranging from ~ 0° to 135°, depending on individual anatomy and activity [17]. Axial rotation occurs to a limited extent when the knee is flexed, while minor lateral movements (abduction/adduction) may occur under specific conditions as passive responses to external forces. However, existing methods for assessing joint torque, including isokinetic dynamometry [18], and inverse dynamics models [19], are confined to laboratory settings, or require complex motion capture setups, limiting their feasibility for real-world applications. The direct measurements like piezoelectric [20] and piezoresistive sensors [21] are invasive, having only one degree of freedom, and fail to comprehensively evaluate its multifaceted biomechanics, thus necessitating the development of advanced wearable sensors capable of capturing these interactions.

Recent advances in wearable technologies have provided encouraging non-invasive approaches, such as surface electromyography (sEMG) [22, 23] and force myography (FMG) [24, 25], that have been used to measure the effective torque at the joints. However, EMG reflects neural activation rather than direct muscle force and is prone to artefacts, while FMG only captures surface-level muscle expansion of all muscles with agonist and antagonist muscle pairs. Ultrasound techniques [11, 26] allow muscle-specific measurements with reasonably good precision, but their bulky form factor and high power consumption make them impractical for long-term wearable monitoring. A close comparison of recent advancements in joint monitoring (Table S1) further underscores the need for a new approach that provides precise, cost-effective, and efficient joint torque assessment, while also enabling long-term rehabilitation monitoring in real-world settings.

Wearable piezoelectric sensors present a promising alternative for real-time biomechanical monitoring [27]. To be suitable for dynamic joint motion sensing, piezoelectric materials must exhibit high sensitivity, flexibility, and stretchability. Boron nitride nanotubes (BNNTs) have emerged as a novel class of piezoelectric nanomaterials with unique advantages for wearable applications due to their exceptional mechanical strength [28], thermal stability [29], and intrinsic piezoelectric properties [30]. BNNTs exhibit high piezoelectric coefficients even at the nanoscale, attributed to the polarization induced by the electronegativity difference between boron and nitrogen atoms. This makes them highly responsive to mechanical deformation, offering a flexible and ultrathin platform for strain-induced charge generation. Previous studies have demonstrated piezoelectric responses in BNNT-polyimide composites [31], BNNT-doped photocurable polymers [32] and polydimethylsiloxane (PDMS)-BNNT composites [33], offering promising routes for fabricating mechanically durable piezoelectric materials. Despite these advantages, integrating high concentration BNNTs into wearable piezoelectric systems remains challenging, primarily due to dispersion difficulties in polymer matrices and the tendency of nanotubes to aggregate, reducing their piezoelectric efficiency. Existing methods, such as surface functionalization and ultrasonic dispersion, improve dispersibility but often compromise nanotube integrity and alignment, limiting their overall performance.

Integrating artificial intelligence (AI) into wearable sensing further enhances the interpretation of joint biomechanics and injury risk assessment [34]. Recent advancements in on-device AI [35–37] have enabled precise motion tracking and real-time data analysis, allowing for more accurate injury prevention and rehabilitation monitoring. However, to be viable for long-term, widespread use, both AI models and wearable devices must operate in low-power (both computational and operational) and resource-limited settings, while ensuring sustainable, continuous monitoring.

**Fig. 1:**
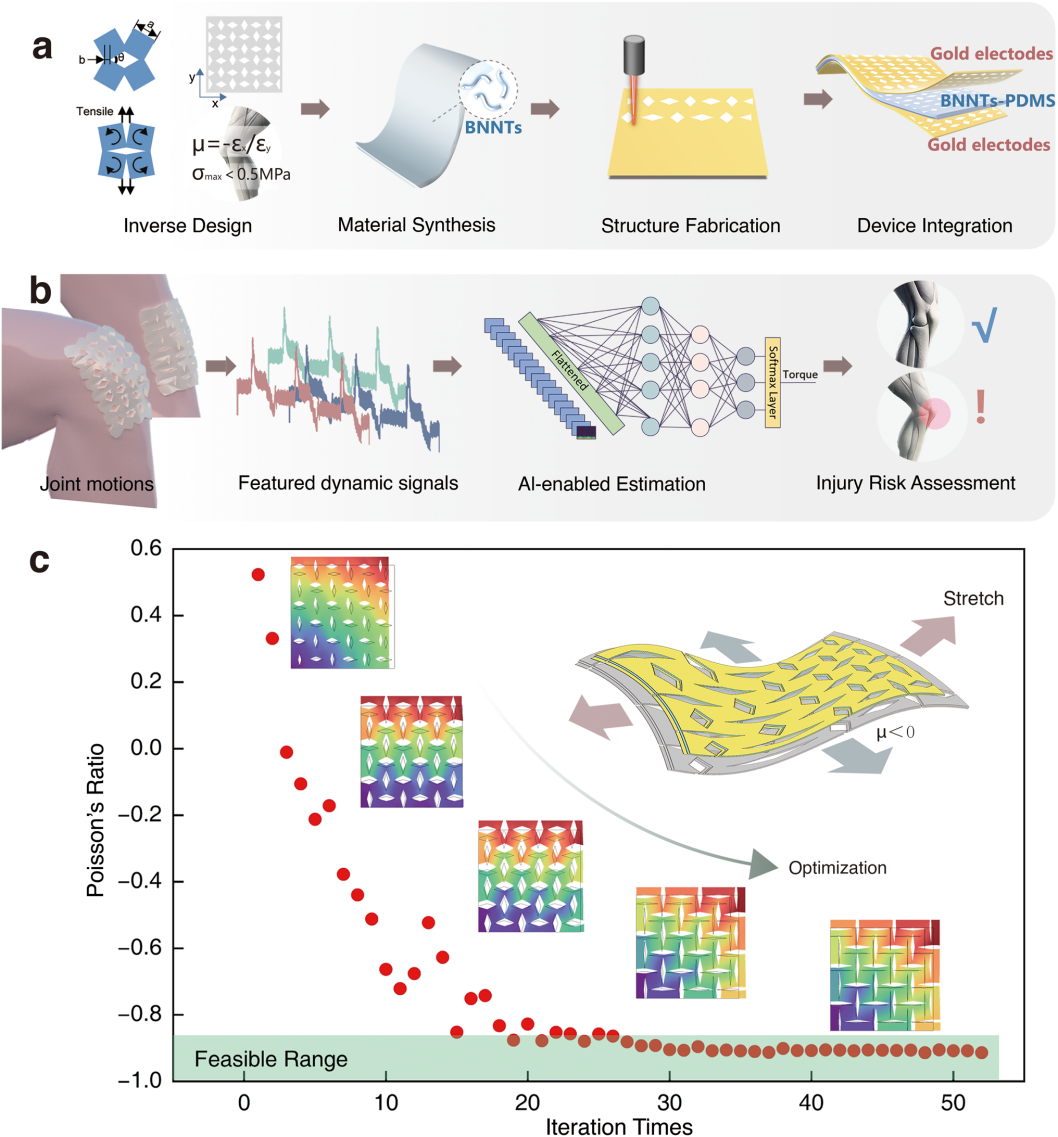
Schematic illustration of AI-assisted knee joint monitoring. **a** Design, synthesis, fabrication, and integration of BNNT/PDMS-based flexible sensors for ergonomic knee adaptation and sensitive dynamic motion capture. **b** AI-assisted estimation of joint injury risk based on dynamic joint signals. **c** Inverse design of the device's auxetic structure to align with knee biomechanics

Herein, we present a new wearable technology for regular joint torque monitoring, utilising piezoelectric BNNTs-based elastomer with embedded on-device AI algorithms. The structure of the piezoelectric elastomer was inversely designed to adapt the flexible and auxetic mechanics of knee joint-specific wearables, while harnessing the unique properties of piezoelectric BNNTs-based elastomers to achieve self-powered sensing, high sensitivity and harvesting complex motion signals at joints (Fig. 1a). A lightweight artificial neural network (ANN) algorithm was employed to analyse complex dynamic motion signals of the knee joint for accurate torque monitoring and to perform the consequent effective risk assessment (Fig. 1b). This technology offers a sustainable solution for long-term joint health monitoring, making it particularly suited for resource-constrained environments, where established healthcare, energy and computational infrastructures are not commonly available. Thus, this AI-enabled, piezoelectric boron nitride nanotubes-based joint-specific wearable can concurrently enable both rapid, effective injury assessment and long-term rehabilitation of joint for diverse populations globally in countries and regions with heterogeneous development levels.

## Materials and Methods

### Material Synthesis

BNNTs with a boron nitride content greater than 99% were purchased from BNNT Materials (United States). To disperse BNNTs in PDMS, a concentration of 12 wt% BNNT relative to the PDMS base was used. Initially, BNNTs were added to tetrahydrofuran (THF) at a 4:1 ratio (THF to PDMS) to reduce viscosity, facilitating dispersion. Manual stirring was initially performed to ensure partial dispersion, followed by vortexing to further break down BNNT clusters. The mixture was vortexed for 24 h and then stirred continuously using a magnetic stirrer for 72 h to achieve uniform dispersion of BNNTs. To prevent BNNT re-clustering during this process, an argon purge was employed to gradually evaporate THF while stirring, a process that lasted approximately 48 h. This slow evaporation allowed the viscosity to increase steadily, ensuring that BNNTs remained homogeneously dispersed throughout the PDMS matrix.

Once dispersion was complete, the BNNT/PDMS mixture was combined with a crosslinking agent at a 1:10 ratio relative to the PDMS base (excluding BNNT content). The final mixture was stirred for 10 min to ensure homogeneous dispersion. For film preparation, the composite was degassed in a vacuum chamber to remove air bubbles. The degassed mixture was placed onto a PET film positioned on a glass plate and cast to a thickness of 1 mm. The cast mixture was left at room temperature for 24 h to cure. Demoulding coatings were applied as needed, depending on the PET sheet properties, to facilitate film removal.

### Laser Cutting and Electrodes Coating

The designed pattern was imported to the laser cutting machine (Trotec, Speedy100R) to fabricate the negative Poisson's ratio (NPR) structure. The film coated with conductive layer was placed in the machine with laser power in 25 W and cutting speed in 1 mm s^−1^. The film was then used to coat electrode layers on both sides. Gold sputtering was performed for 20 min to deposit an ultrathin (~ 500 nm) gold layer. While gold is generally perceived as a high-cost material, the thin film sputtering technique used in our fabrication process significantly reduces material consumption, making it a cost-effective choice. The total gold usage per device is less than 10 mg, and based on current market prices, the estimated material cost remains below $0.80 per device. This makes it comparable to alternative materials like silver, while offering superior long-term stability and performance.

### Tensile Testing

PDMS is mixed with crosslink agent at a 10:1 ratio and spread onto flat glass. It is crosslinked at 80 °C for 3 h to form a thin film. Both PDMS and BNNTs-PDMS films were cut into a dog-bone shape, commonly used for tensile testing, with a gauge width of 8 mm, a gauge length of 20 mm, and a thickness of 0.30 mm. This standardized geometry ensures uniform stress distribution during mechanical testing. At least three samples were tested for each material with a constant displacement rate of 10 mm min^−1^ using Instron 5564. Tensile modulus of each sample was determined within the 20% of the maximum strain and calculated based on the Eq. (1):

1 $$E=\frac{{\sigma }_{l}}{{\varepsilon }_{l}}$$

where ${\sigma }_{l}$ is the maximum stress within the linear tensile range, ${\varepsilon }_{l}$ is the corresponding elastic strain. Toughness of each material was calculated from the area under the nominal stress–strain curve:

2 $$T={\int }_{0}^{{\varepsilon }_{f}}\sigma d\varepsilon$$

where ${\varepsilon }_{f}$ is the strain upon failure, *σ* is the engineering stress and *ε* is the engineering strain.

### Finite Element Analysis

The finite element analysis was carried out with COMSOL Multiphysics. A step displacement was applied to the NPR [38–40] model formulated in COMSOL, whose Poisson’s ratio and von Mises stress were calculated to evaluate the performance of the model design. The BNNTs/PDMS material parameters were set up as tensile testing results which are Young’s modulus *E* = 0.63 MPa and Poisson’s ratio *μ* = 0.20, assuming a linear elastic response. The tensile process of the patch in general usage is within the low frequency (< 10 Hz) and elastic range, the structure is considered as linear elastic material during the finite element analysis. The initial structure was meshed in quadratic triangular elements and then optimised with tensile displacement of 10 mm and film thickness of 0.30 mm. During the optimization, Nelder-Mead simplex algorithm [41] was adopted as shown in Fig. S1f, in which the judgement condition *δ* = 0.01. Structure parameters were regulated within ranges shown in Fig. S1c. The optimised structure was then calculated with displacement from 2 to 10 mm to analyse its influence on the Poisson’s ratio.

### Materials Characterization

The crystalline structures of the nanocomposites were characterized using X-ray diffraction (XRD) with a Rigaku MiniFlex system, utilising a Cu Kα radiation source (*λ* = 1.541 Å). Atomic force microscopy topography and nano-FTIR spectra of the nanocomposites were obtained using a scattering-type scanning near-field optical microscope (Neaspec s-SNOM). The measurements were performed under tapping mode using a platinum-coated atomic force microscopy (AFM) tip (~ 20 nm radius, NanoAndMore) oscillating at 250 kHz, while being illuminated by a broadband femtosecond infrared laser (TOPTICA Photonics) [42]. Fourier-transform infrared (FTIR) spectra were collected using a Nicolet iS10 spectrometer with an attenuated total reflectance (ATR) module. For scanning electron microscopy imaging, samples were coated with a thin layer of Au/Pd and observed using a Zeiss EVO microscope.

### Standard Piezoelectric Output Testing

The prepared BNNT/PDMS was prepared as a sandwiched by two copper electrodes for 2.0 cm × 2.0 cm standard device and tested in compression mode using a vibrational punch with a surface area of 1.13 cm^2^. The compression force was applied using a magnet shaker (Brüel & Kjær LDS V201) driven by a voltage-amplified arbitrary function generator (GW Instek AFG-2105). The output voltage was recorded using an oscilloscope (Rigol DS1054Z), while the output current of the device was measured using an electrometer (Keithley 6514). The maximum power density (*P*) was determined using the formula:

3 $$P=\frac{{V}^{2}}{A\cdot R}$$

where *V* is the voltage measured across a resistance load *R*, *A* denotes the nominal contact area of the device.

### Data Processing and Machine Learning Model

Machine learning dataset was collected and processed using Arduino Nano 33 BLE equipped with an ADC module for voltage measurement. At least 100 motion data were collected from each movement. Pre-Processing Pipeline: The average duration of recorded data for each experimental condition was 5 min, sampled at a frequency of 1000 Hz. The pre-processing procedure consisted of three steps: (1) Segmentation: The subject was instructed to perform knee flexion exercises every 3 s during data recording, and the signals were segmented accordingly, resulting in 1056 segments. (2) Short-Time Fourier Transform (STFT) spectrum [43]: Each segment was subjected to an STFT with a window size of 256 data points and 50% overlap, using Python's SciPy library. (3) Feature Extraction: The mean frequency components of the STFT were computed to serve as input features for model training.

To improve the robustness of joint torque estimation, multiple noise reduction techniques were implemented. White noise was filtered during preprocessing, while physiological noise from heart rate and respiration was attenuated using STFT filtering to remove low frequency artefacts. Mechanical noise, caused by sensor instability or sudden impacts, was minimized by securing the sensor with medical tape and leveraging frequency domain filtering to suppress transient disturbances. To enhance model generalization, cross-validation and data washing were applied to mitigate overfitting, while a fine-tuning process allowed the model to adapt to new users with minimal additional data. These strategies ensured stable signal acquisition and improved the reliability of real-time torque estimation across different movement conditions. All models were implemented and trained using TensorFlow (version 2.10.0), running on Python 3.7.12. Three models were developed:

#### Baseline Model Based on Standard Knee Torque Dataset

The dataset was divided into training, validation, and test sets in a 7:1:2 ratios. An ANN with two dense layers was developed to analyse the features from different knee torque. By adding and adjusting a third dense layer, the model can be tailored to achieve different objectives, such as classification to identify discrete torque categories or regression to estimate continuous torque values.

#### Classification Based Knee Torque, Angle, and Load Estimation Model

The knee torque estimation model was developed using a baseline neural network architecture. The dataset for this model was labelled with nine distinct torque levels, enabling the third dense layer to output probabilities corresponding to these 9 categories. A softmax layer was appended to normalize the probabilities, facilitating the classification of knee torque into one of the predefined levels. The model was trained using the Adam optimizer, with the sparse categorical cross-entropy loss function employed to evaluate classification error. Accuracy was defined as the proportion of correctly predicted labels relative to the total number of samples. The model was trained for 100 epochs, allowing sufficient exposure to the dataset for effective feature learning. The knee angle and load estimation models were developed using a similar methodology. These models utilised datasets labelled with knee angle and load categories, respectively, with the third dense layer configured to output probabilities for 3 distinct classes. This consistent approach ensured that the models were capable of accurately classifying knee biomechanical parameters based on their respective datasets.

#### Regression Based Knee Torque, Angle and Load Estimation Model

The regression model was developed to predict continuous knee torque values based on the extracted features. Building upon the baseline architecture, the third dense layer of the model was configured with a single output neuron, corresponding to the continuous torque value. The model was trained using the Adam optimizer, which adapts the learning rate during training to achieve efficient convergence. The mean squared error (MSE) was used as the loss function to measure the squared differences between predicted and true torque values. The training process was conducted over 100 epochs. The angle and load estimation models employed the same structure as the torque estimation model. These models were trained using datasets labelled with angle and load, respectively. The Pearson correlation values were computed using the formula:

4 $$r= \frac{\sum ({X}_{i}- \overline{X })({Y}_{i}- \overline{Y })}{\sqrt{\sum ({X}_{i} - \overline{X }{)}^{2}} \cdot \sqrt{\sum ({Y}_{i}- \overline{Y }{)}^{2}}}$$

where r represents Pearson correlation coefficient, X represents the predicted value, and Y represents the true value.

### Computational Efficiency of the Model

The computational efficiency of the model was analysed by measuring the model size and the required floating point operations. To further evaluate the model efficiency, the models were subsequently converted into TensorFlow Lite format and deployed on the STM32 microcontroller (NUCLEO F401RE, ARM 32-bit Cortex-M4 CPU) using STM32CubeIDE (version 1.16.0) with X-Cube-AI extension (version 9.0.0). Post-deployment evaluation was conducted using X-Cube-AI to assess the performance and resource utilization of the deployed model.

### Real Time Monitoring and Evaluation

The real-time monitoring system employs a knee torque classification model, integrated into a MATLAB application, to evaluate knee biomechanics in real-time. To ensure reliability under real-world conditions, where knee torque may occasionally be absent, the dataset includes instances of zero torque, enabling the model to accurately predict such states. The system features a graphical user interface (GUI) that provides real-time visualization of knee torque values. Sensor data is transmitted to the application via universal asynchronous receiver/transmitter (UART) communication and displayed on dedicated graphical axes within the GUI. The system continuously monitors knee torque, generating warnings and delivering medical advice if the measured torque exceeds predefined safety thresholds. The incoming piezoelectric responses are stored in a circular data buffer with a capacity of 3000 data points, corresponding to approximately 3 s of real-time data. This buffer dynamically updates to retain only the most recent 3000 data points, ensuring the system always operates with up-to-date information. Data processing occurs every 3 s, during which the system extracts the latest dataset from the buffer for analysis. The extracted data are processed using STFT to convert the time domain signals into the frequency domain. The mean value of the frequency spectrum is calculated and used as an input feature for the classification model. The pre-trained model, originally developed in Python and exported in TensorFlow format, is imported into MATLAB using the Deep Learning Toolbox Converter for TensorFlow Models (version 24.1.0). Predictions generated by the model, along with raw sensor data, are systematically recorded and stored in a CSV file for further analysis. This robust pipeline ensures accurate, real-time evaluation of knee torque, while providing actionable insights and warnings to users in dynamic conditions.

## Results

### Inverse Structure Design of the Flexible Wearable Device Based on Knee Biomechanics

The knee joint's complex load-bearing structure and dynamic motion present significant challenges for accurate monitoring, particularly under complex loading conditions such as flexion, extension, and rotation. Recent advancements in wearable joint monitoring devices have explored a variety of structural configurations to track complex joint movements, as shown in Table S2. Common designs include hinged systems, such as knee braces with textile-based sensors embedded in circular hinge sections, and devices with tensile “ligament” structures coupled with rotary position sensors at pivot points. These are often designed with holders near the knee joint that allow adaptation to bending motions and are optimised for capturing rotational data. Another prevalent approach uses strap-based devices, such as inertial or magneto-inertial measurement units worn on the ankle or thigh, or capacitive sensor systems worn as skin patches. These systems are typically suited for three-dimensional motion analysis but rely on external attachment, which can limit mechanical coupling with the joint itself. To address these challenges, our device employs an inverse design strategy tailored to the biomechanics of the knee joint, incorporating an auxetic design. By conforming to the natural flexion, extension, and rotational movement patterns, the flexible structure of our device ensures a closer mechanical match to the skin-joint interface. This not only improves motion tracking fidelity but also enables more detailed sensing of complex loading conditions, offering enhanced signal resolution for dynamic joint analysis.

Auxetic materials, characterized by a NPR, expand laterally when stretched and contract laterally when compressed, contrary to conventional materials whose Poisson’s ratio (*μ*) is bounded by the limits of (fully compressible) 0 ≤ *μ* ≤ 0.5 (incompressible). This unique mechanical behaviour allows auxetic structures to conform more effectively to the dynamic surface of the knee, ensuring consistent contact and flexibility during movement. A rotational square pattern was selected as the fundamental unit of the structure. Three structure parameters were optimised to satisfy the requirement on Poisson’s ratio and material ultimate stress as shown in Fig. 1a, in which *a* is the length of the square, *b* is the joint width between squares, and* θ* is the original angle between squares. The Nelder-Mead method was used to optimize the whole structure automatically, of which the target function is defined as following Eq. (5):

5 $$f\left(\mu , \sigma ;a,b,\theta \right)=\left\{\begin{array}{l}\mu +1, \sigma \le 0.5MPa\\ \mu +11, \sigma >0.5 MPa\end{array}\right.$$

where *μ* is the Poisson’s ratio and *σ* is the maximum von Mises stress of the whole structure. As shown in Fig. 1c, the optimization process aimed to minimize the target value to approach 0, resulting in *μ* approaching − 1, consistent with the behaviour of human skin under 10% strain. When the maximum stress is larger than 0.5 MPa, an extra 10 will be added to the whole target as a penalty. Figure S1a-c illustrates three examples of structures with different structural parameters during the optimization. The patterned patch finite element model was set to be stretched along the length direction for 10 mm, whose Poisson’s ratio was defined as:

6 $$\mu =-\frac{({W}_{t}-{W}_{i})/{W}_{i}}{({L}_{t}-{L}_{i})/{L}_{i}}$$

where ${W}_{i}$ and ${L}_{i}$ are initial width and length of the patch model respectively, while ${W}_{t}$ and ${L}_{t}$ are the width and length of that after tensile deformation. The material was modelled as a linear elastic material, with the maximum deformation remaining within its linear elastic range. It is worth noting that the stress concentration at the corners can compromise the structural integrity of the device (Fig. S1b), while rounding these corners may alter its negative Poisson’s ratio properties. Therefore, the parameter was carefully optimised to ensure that the maximum stress remained below 0.5 MPa. The optimization process based on the Nelder-Mead algorithm was performed over 40 iterations to achieve the targeted Poisson’s ratio, as shown in Fig. S1d-f. The optimised result yields a Poisson’s ratio in the range of -0.94 with the optimised parameters of *a* = 10 mm, *b* = 3 mm and *θ* = 175°. The parameter was then used in the fabrication of the flexible device for a more effective dynamic contact.

### Design and Characterization of Elastic Composites Based on Boron Nitride Nanotubes and Poly(dimethylsiloxane)

**Fig. 2:**
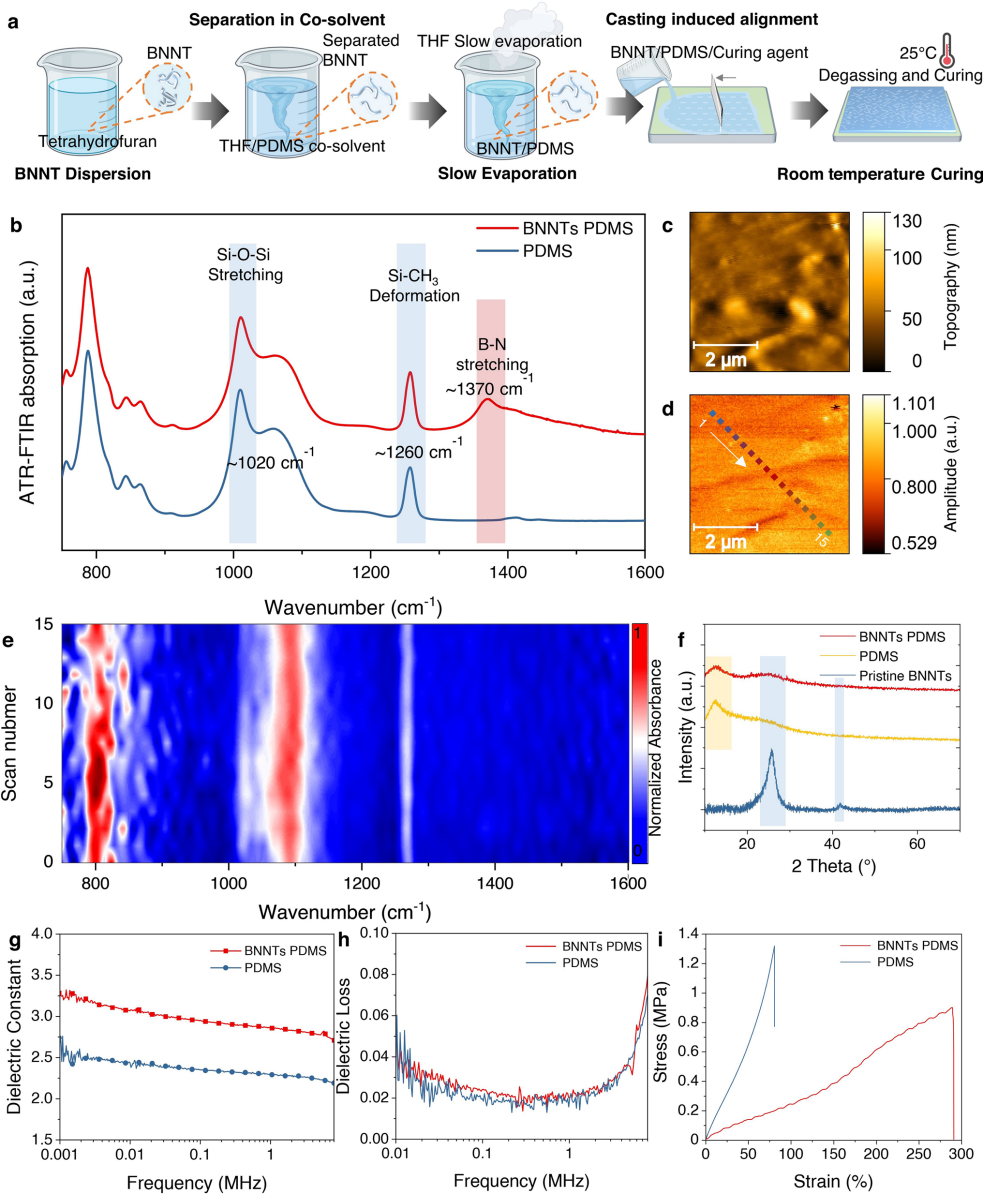
Fabrication and characterization of flexible BNNTs/PDMS film. **a** Fabrication of BNNTs/PDMS film with uniform dispersion of nanotubes. **b** ATR infrared absorption of BNNTs/PDMS. **c** Topography and **d** s-SNOM infrared image of near field optical amplitude (O2A signal) of BNNTs/PDMS film (The points of the line scans are coloured). **e** NanoFTIR absorption spectra of BNNTs/PDMS surface taken from the coloured line scans defined in **c**, with a spatial resolution of ≈20 nm [42]. **f** X-ray diffraction of BNNTs/PDMS. **g** Dielectric constant and **h** dielectric loss of BNNTs/PDMS. **i** Strain–stress curve of BNNTs/PDMS

BNNTs with a purity of nearly 99% boron nitride content were utilised in this study. As shown in Fig. 2a, BNNTs were prepared at a concentration of 12 wt% relative to the PDMS base. BNNTs exhibit piezoelectric properties due to their non-centrosymmetric structure, making them highly desirable for enhancing charge generation in piezoelectric composites. Ideally, a higher BNNT content improves the piezoelectric response; however, achieving both high loading and uniform dispersion is extremely challenging due to the strong van der Waals interactions and high aspect ratio of BNNTs, which promote aggregation. The BNNTs were pre-dispersed in THF without modification and vortexed to break up initial BNNT clusters (THF in a 4:1 mass ratio to PDMS). This mixture was then added to the PDMS base and gently stirred for 48 h to ensure uniform dispersion of the BNNTs. THF was gradually evaporated under a nitrogen flow, while stirring until only one-fifth of the original volume remained, effectively preventing nanotube re-clustering. The BNNT mixture was then combined with a crosslinking agent and curing agent in a 10:1 mass ratio. For casting, a doctor blade was gently used to spread the PDMS in a single direction, to promote the induced alignment of the BNNTs. It is worth noting that our fabrication method introduces a facile casting process that mechanically induces BNNT arrangement in plane, eliminating the need for additional poling treatments. Traditional piezoelectric materials typically require high-voltage poling to align dipoles and activate their piezoelectric properties. However, our method leverages controlled casting-induced arrangement, which enables self-organization of BNNTs in a manner that enhances piezoelectric efficiency without extra processing steps. This not only simplifies fabrication but also reduces energy consumption and production costs, making the approach more scalable.

The uniform dispersion of BNNTs in the PDMS matrix was confirmed through a series of characterization techniques, as shown in Fig. 2. Our approach successfully disperses BNNTs throughout the matrix without compromising their alignment or functionality, as evidenced by both macroscale ATR-FTIR spectrograms and nanoscale nano-FTIR measurements. ATR-FTIR spectroscopy (Fig. 2b) was employed to analyse the chemical interactions between BNNTs and the PDMS matrix. Both BNNTs/PDMS and pure PDMS exhibit characteristic peaks at 1020 cm⁻^1^ (Si–O–Si stretching) and 1260 cm⁻^1^ (Si–CH_3_ deformation), indicating that the intrinsic structure of PDMS is preserved in the composite. Additionally, the BNNT/PDMS film shows a distinct absorption peak at ~ 1370 cm⁻^1^, corresponding to B–N stretching vibrations, confirming the successful incorporation of BNNTs into the matrix without significant chemical modification or aggregation. The pristine BNNTs were also characterized using near field s-SNOM infrared imaging, as shown in Fig. S2a-c. The nanotubes observed as uniformly dispersed cylindrical fibres, were isolated to approximately 50 nm in width but extending to lengths of several micrometers, exhibited distinct characteristic absorption peaks at ~ 1370 cm⁻^1^ at the nanoscale.

In Fig. 2c, topographical imaging using AFM reveals a smooth and uniform surface morphology of the composite film, indicative of well-dispersed BNNTs throughout the matrix. This observation is further supported by s-SNOM infrared amplitude mapping (Fig. 2d), using the second-harmonic near field optical signal O2A, which displays consistent PDMS with less distinct features corresponding to nanotubes. The line scans, contour plotted for spatial correspondence, demonstrate localized BNNT distribution in PDMS with a spatial resolution of less than 20 nm. Nano-FTIR absorption spectra (Fig. 2e) primarily show peaks associated with PDMS, reflecting its dominant presence in the matrix. The clean and uniform BNNTs/PDMS surface observed by scanning electron microscopy and optical stereo transmission microscopy further supported the uniformity of PDMS, as shown in Fig. S2d. It is worth to note that localized peaks at 1370 cm⁻^1^, corresponding to B–N stretching, can also be observed in certain point scans (Fig. S2e), confirming the nanoscale integration of BNNTs within the composite. XRD patterns (Fig. 2f) display sharp peaks characteristic of BNNTs, including the (002) reflection located at approximately at 2θ of 25.9° and the (100) reflection near 41.7°. The broad peak observed around 12.3° in the XRD pattern of PDMS and the composite material reflects its amorphous nature, attributed to the short-range order of its silicon-oxygen (Si–O) backbone. The presence of the BNNTs characteristic peaks in the BNNTs/PDMS composite verifies that the structure of BNNTs is well preserved in the PDMS matrix. The PDMS matrix itself is amorphous and could overlap or interfere with the BNNT peaks leading to broadening of the characteristic peaks.

Dielectric measurements demonstrate enhanced electrical properties in the BNNT/PDMS composite compared to pure PDMS (Fig. 2g, h). For instance, the dielectric constant increases from approximately 2.29–2.86 at 1 MHz, indicating effective polarization induced by the BNNTs. Despite this enhancement, the dielectric loss remains low, signifying minimal energy dissipation and making the composite suitable for flexible electronics. The stress–strain curve (Fig. 2i) highlights the typical mechanical improvements especially in the ductility of the BNNTs/PDMS composite compared to pure PDMS. At least three samples (n = 3) were tested for quantification of the mechanical properties of BNNTs/PDMS composites (Fig. S3a, b). The maximum strain increases from 72.0 ± 10.0% in pure PDMS to 223.8 ± 48.4% in BNNTs/PDMS, demonstrating enhanced ductility and flexibility due to the uniform dispersion of BNNTs. Notably, PDMS is characterized by brittle fracture behaviour, where cracks propagate rapidly under stress, leading to sudden failure. In contrast, BNNTs/PDMS exhibit a more ductile fracture mechanism, with significantly slower crack propagation and an increased ultimate strain. The toughness, representing the energy absorption before failure, increases from 37.8 ± 8.3 J cm^−3^ in pure PDMS to 81.9 ± 30.9 J cm^−3^ in BNNT/PDMS, reflecting the synergistic interaction between the nanotubes and the matrix. BNNTs distribute applied stresses evenly, reducing stress concentrations and enhancing the material's ability to elongate under load. Their integration improves energy absorption by delaying crack propagation and allowing efficient stress transfer at the BNNT-PDMS interface. Additionally, BNNTs stabilize the matrix during deformation, preventing premature failure and enabling greater flexibility. These synergistic effects between the elastic PDMS and mechanically robust BNNTs result in a composite with significantly improved ductility and toughness, ideal for applications requiring both stretchability and durability. However, the tensile modulus decreases from 1.6 ± 0.1 to 0.6 ± 0.1 MPa. The incorporation of BNNTs can affect the crosslinking density of the PDMS matrix by physically disrupting the polymer network, leading to reductions in the stiffness of the composite film. Collectively, the uniform and high concentration loading of BNNTs in the PDMS matrix demonstrated improved mechanical and dielectric properties, making them highly suitable for further flexible and durable piezoelectric wearable devices.

### Electrical Performances of the Device Based on BNNTs/PDMS

**Fig. 3:**
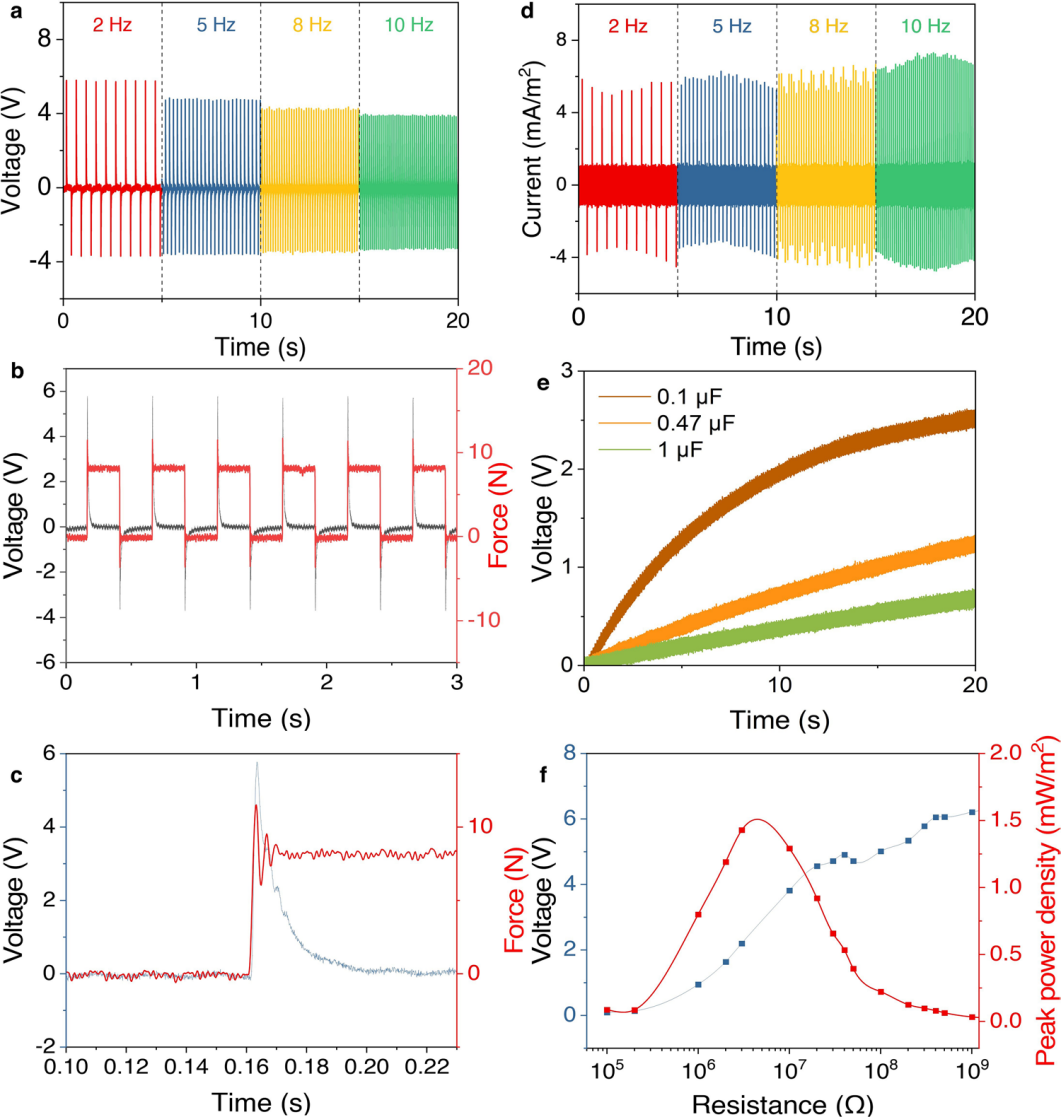
Piezoelectric output of BNNTs/PDMS under standard loading conditions. **a** Open-circuit voltage output of a BNNTs/PDMS film (12 wt% BNNTs, 2 cm × 2 cm) under cyclic compression loading at frequencies of 2, 5, 8, and 10 Hz. **b** Voltage output of the BNNTs/PDMS film under cyclic loading forces. **c** Enlarged view of **b**, showing the detailed relationship between loading and voltage over 0.1 s. **d** Short-circuit current of the BNNTs/PDMS film under cyclic compression loading at 2, 5, 8, and 10 Hz. **e** Charging curves of capacitors (0.1, 0.47, and 1 μF) over 20 s under 2 Hz cyclic compression loading of the piezoelectric BNNTs/PDMS film. **f** Closed-circuit peak voltage and power density across a loading resistor powered by the BNNTs/PDMS film under a constant input frequency of 2 Hz and a peak compression load of 11.5 N

To evaluate the capability of the BNNTs/PDMS material for use as responsive sensor to capture the dynamic knee motions, the piezoelectric performance of the BNNTs/PDMS film under standard loading conditions was evaluated to demonstrate the performance of the piezoelectric material. To examine the effect of BNNTs content on piezoelectric performance, composites with 0, 4, 8, and 12 wt% BNNTs were fabricated and tested. As shown in Fig. S3c, the voltage output increases systematically with higher BNNT loading, attributed to the enhanced charge generation and stress transfer facilitated by BNNTs, as PDMS itself is non-piezoelectric. However, beyond 12 wt%, agglomeration due to strong van der Waals interactions disrupts uniform dispersion, hindering effective stress distribution and reducing performance. Therefore, 12 wt% was identified as the optimal concentration, balancing high piezoelectric sensitivity with uniform dispersion and film integrity. A thin film of 2 cm × 2 cm was used in the standard test under compression load, while the performances are illustrated in Figs. S4 and 3. The open-circuit voltage output of the BNNTs/PDMS film under cyclic compression at various frequencies demonstrates a clear frequency-dependent response, as shown in Fig. 3a. The electrical performances are statistically quantified using mean ± standard deviation derived from five tested cycles. At a frequency of 2 Hz, the film produces a voltage of 5.76 ± 0.02 V, which slightly decreases to 4.74 ± 0.03 V at 5 Hz, 4.275 ± 0.01 V at 8 Hz, and 3.89 ± 0.01 V at 10 Hz. This reduction in voltage with increasing frequency can be attributed to shorter compression cycles at higher frequencies, resulting in reduced charge accumulation on the surface of the film. The data confirm the consistent piezoelectric response of the composite under low frequency loadings, which covers most of the knee motions (< 10 Hz). The relationship between voltage and applied force is linear and consistent, with a sensitivity of 0.50 ± 0.01 V N^−1^. Compared to existing BNNT- or PDMS-based piezoelectric films (Table S3 and Fig. S5), the reported BNNTs/PDMS nanocomposite exhibits enhanced sensitivity. The improved performance can be attributed to the high BNNT loading (12 wt%), which increases the density of active piezoelectric domains, thereby enhancing charge generation under mechanical deformation. Additionally, the uniform dispersion of BNNTs within the PDMS matrix ensures effective stress transfer, reducing localized charge screening effects and maximizing the overall piezoelectric response. The intrinsic piezoelectricity of BNNTs, driven by the polarization induced by the electronegativity difference between boron and nitrogen atoms, is further amplified by their in plane arrangement, which facilitates directional charge accumulation.

The consistency across different forces highlights the film’s potential for precise sensing applications (Fig. 3b). A detailed analysis over 0.1 s reveals a typical piezoelectric behaviour (Fig. 3c), where the voltage increases with the applied force. Voltage is generated during the loading phase and returns to zero during unloading, further validating the dynamic response of the elastic BNNTs/PDMS film. This characteristic confirms the potential of the material for real-time force monitoring in applications requiring dynamic load sensing. As shown in Fig. 3d, the short-circuit current output of the film increases with frequency, demonstrating values of 5.516 ± 0.25 mA m^−2^ at 2 Hz and 6.9 ± 0.42 mA m^−2^ at 10 Hz. The generated current was normalized with the area under the loading curve. The higher current at increased frequencies is consistent with the enhanced flow of charge carriers due to the repeated deformation of the composite. This robust current output reflects the efficient charge transfer properties of the BNNTs/PDMS film under cyclic compressive loading. The self-powering capability of the BNNTs/PDMS film was evaluated by charging commercial capacitors of different capacitances (0.1, 0.47, and 1 μF) under a steady 2 Hz compression load of 12 N (Fig. 3e). Notably, the 0.1 μF capacitor was charged to 2.5 V within 20 s, showcasing the film’s ability to effectively convert energy during mechanical deformation. A maximum power output of 1.47 mW m^−2^ was achieved when a 3 MΩ resistor was applied under a constant input frequency of 2 Hz and a peak compression load of 11.5 N (Fig. 3f). This demonstrates the composites’ efficiency in converting mechanical energy into electrical energy and the potential for self-powered sensing in wearable device.

To assess the mechanical durability of the device, we conducted continuous cyclic loading tests for 30,000 cycles under standardized conditions, as shown in Fig. S6a. The sensor exhibited a stable piezoelectric output throughout the cycles, with negligible performance degradation, indicating its long-term reliability. Additionally, SEM imaging was performed after the durability test (Fig. S6c) to examine potential structural damage. The post-test surface morphology remained consistent with that of newly prepared BNNTs/PDMS samples, with no visible cracks or deterioration, further confirming the mechanical integrity of the composite. Furthermore, the influence of humidity on-device performance was investigated to evaluate its stability under practical environmental conditions (Fig. S6b). The experiment was conducted in a controlled glove bag environment, where relative humidity (RH) was precisely adjusted by purging dry nitrogen gas to decrease RH and introducing humidified air to increase RH. The results showed that the output voltage remained stable at RH levels below 75%, attributed to the hydrophobic nature of the PDMS matrix, which effectively prevents moisture absorption. However, when RH exceeded 75%, a gradual decrease in output voltage was observed, likely due to increased charge dissipation caused by moisture adsorption. Given that ambient humidity in daily environments typically ranges from 30% to 70%, the device is expected to maintain reliable performance under normal operating conditions. To further enhance humidity resistance for future applications, additional strategies such as hydrophobic polymer encapsulation and surface functionalization with water-repellent coatings are being explored to prevent direct moisture exposure and improve long-term stability in high-humidity environments.

### Monitoring and Evaluation of the Dynamic Motions at the Knee Joint Based on Machine Learning Model

Combining the inversely designed wearable structure and the highly sensitive BNNTs/PDMS material, we fabricated a wearable device that is tightly attachable on the knee joint during movement, while able to collect the very detailed and complex motion signals during dynamic knee activities. The responsive motion signal of the device attached to the knee joint would generate electric signals under complex loading of flexion, extension, and rotation during knee motions. The responsive data was used to train a machine learning model for evaluating knee bending angles, loading conditions and further estimating torque applied to the knees.

**Fig. 4:**
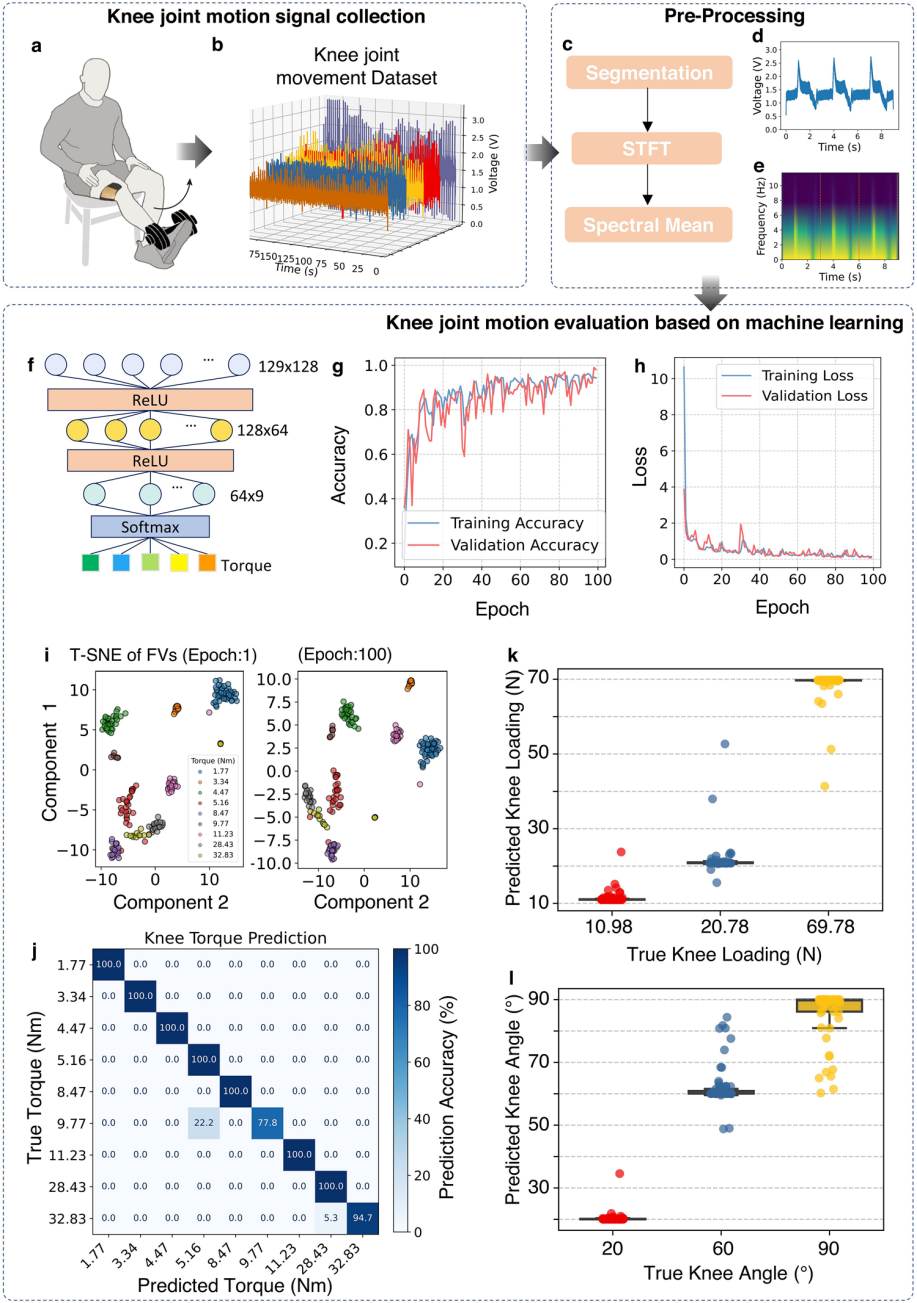
Classification based model for monitoring knee joint activities. **a** Illustration of the participant performing knee flexion with the device attached on the knee joint and collected dataset. **b** Featured signals in response to different motions collected by the flexible device worn by the participant. **c** Preprocessing pipeline for the knee joint motion signal. **d** Example segmented data recording knee bending under a 9.8 N loading. **e** STFT spectrum of the example data. **f** Structure of the classification model. **g** Training and **h** validation accuracy plot over 100 epochs. **i** Feature vector matrix at initial (epoch:1) and end (epoch:100) of the processing by the T-SNE algorithm. **j** Confusion matrix of the prediction accuracy of different knee joint torques. **k** Prediction of knee loadings based on the trained model. **l** Prediction of the knee bending angles based on the trained model

To train a baseline model for mapping the relationships between the knee motions and the correspondence signal features, we collected a database in a well-controlled lab environment. The device was securely attached to the knee joint, with one end fixed at the bottom of the quadriceps femoris muscle, where it transitions into the tendon, and the other end fixed to the patellar tendon, ensuring stable positioning and optimal contact for accurate detection of knee motion and signal acquisition during joint activities (Fig. 4a). During the data collection, the participant was seated on a bench, performing knee flexion exercise using a dumbbell with the device attached on the side of the thigh (Fig. S7a). Data were collected under nine conditions to predict the torque. These conditions included three loading levels (0, 9.8, and 58.8 N) and three knee joint angles (20°, 60°, 90°), resulting in nine combinations. Each condition was repeated 100 times for a training database. The featured signals (Fig. 4b) reveal distinct response patterns corresponding to different motions, with several small motion-related features emerging prominently. While these features highlight the sensitivity of the device to subtle movements, some remain unexplainable, thus required machine learning algorithms to gain further insights. A preprocessing pipeline (Fig. 4c) was applied, including segmentation and normalization. An example of segmented data under a 9.8 N mass (Fig. 4d) highlights the motion-specific signal characteristics, while the STFT (Fig. 4e) visualizes the time–frequency components critical for the motion features.

The features were trained through three dense layers and two rectified linear unit (ReLU) layers (Fig. 4f) for classification of biophysical features embedded in the electrical signals. The accuracy and loss of the training and testing reached peaks up to 20 epochs, reflecting the data quality and robust model performance, the consistent trends in accuracy and loss between the training and validation datasets demonstrate no overfitting during model training, as shown in Fig. 4g, h. T-distributed stochastic neighbourhood embedding (T-SNE) visualization [44] of feature clustering (Fig. 4i) shows significant improvement in class separability from the initial epoch to the final epoch of 100.

To establish a connection between the biophysical mechanics of the motions and the corresponding responsive signals, the collected participant data were utilised to estimate the torque generated during the specified standard movements. The knee torque was calculated using following equation [45]:

7 $$\tau = F \times L \times {\text{ sin}}\left( \theta \right)$$

where *τ* represents the joint torque, *F* represents the load on the knee, which is determined by the combined weight of the foot and the external load, *L* represents the distance measured from the knee joint to the ankle, and *θ* represents the angle of the lower leg relative to the vertical direction. The foot weight was estimated as 1.4% of the participant's body weight, which equaled 10.98 N, and the measured distance was 0.47 m. The torque values corresponding to each experimental condition are shown in Fig. S7b. In each experimental condition, the typical outputs in the time domain and frequency domain can be found in Figs. S8 and S9, respectively.

The model was then used to estimate the torque level applied to the knee joint during movement based on the standard dataset. The confusion matrix (Fig. 4j) confirms high classification accuracy across different torque levels, the testing accuracy is 97.5%, validating the model's precision in estimating the torque level applied. Furthermore, the predictions of knee loadings (Fig. 4k) and bending angles (Fig. 4l) show excellent agreement with the ground truth data, reflecting the system's high reliability in evaluating joint biomechanics. To obtain continuous data using classification model, the probability value from the third dense layer was derived. The predefined numerical labels were taking a weighted sum using predicted probabilities as weights to obtain a continuous value. The root mean square error (RMSE) quantifies the average prediction errors, calculated as the square root of the mean of squared differences between predicted and actual values. In this study, the RMSE values for knee joint torque prediction is 2.45 Nm. The knee loading and bending angle prediction model has also been trained by changing the dataset label from torque to loading and angle respectively. For the knee loading and bending angle predictions in this study, RMSE values of 4.87 N and 5.36°, respectively, suggesting that the model effectively captures the underlying biomechanical patterns with minimal deviation from the actual values. Furthermore, the proposed models achieved both high accuracy and computational efficiency, making them suitable for deployment on resource-constrained edge devices commonly used in wearable technology, such as STM3 microcontrollers and Texas Instruments MSP430 microcontrollers. This enables real-time, localized data processing with minimal latency, while preserving the overall wearability and practicality of the device. The architecture of the knee torque prediction model consists of 75,017 parameters, requiring less than 0.29 MB of memory for storage. In terms of computational complexity, the model performs approximately 149,622 floating point operations during a single inference pass and the average inference time was recorded as 6.870 ms.

**Fig. 5:**
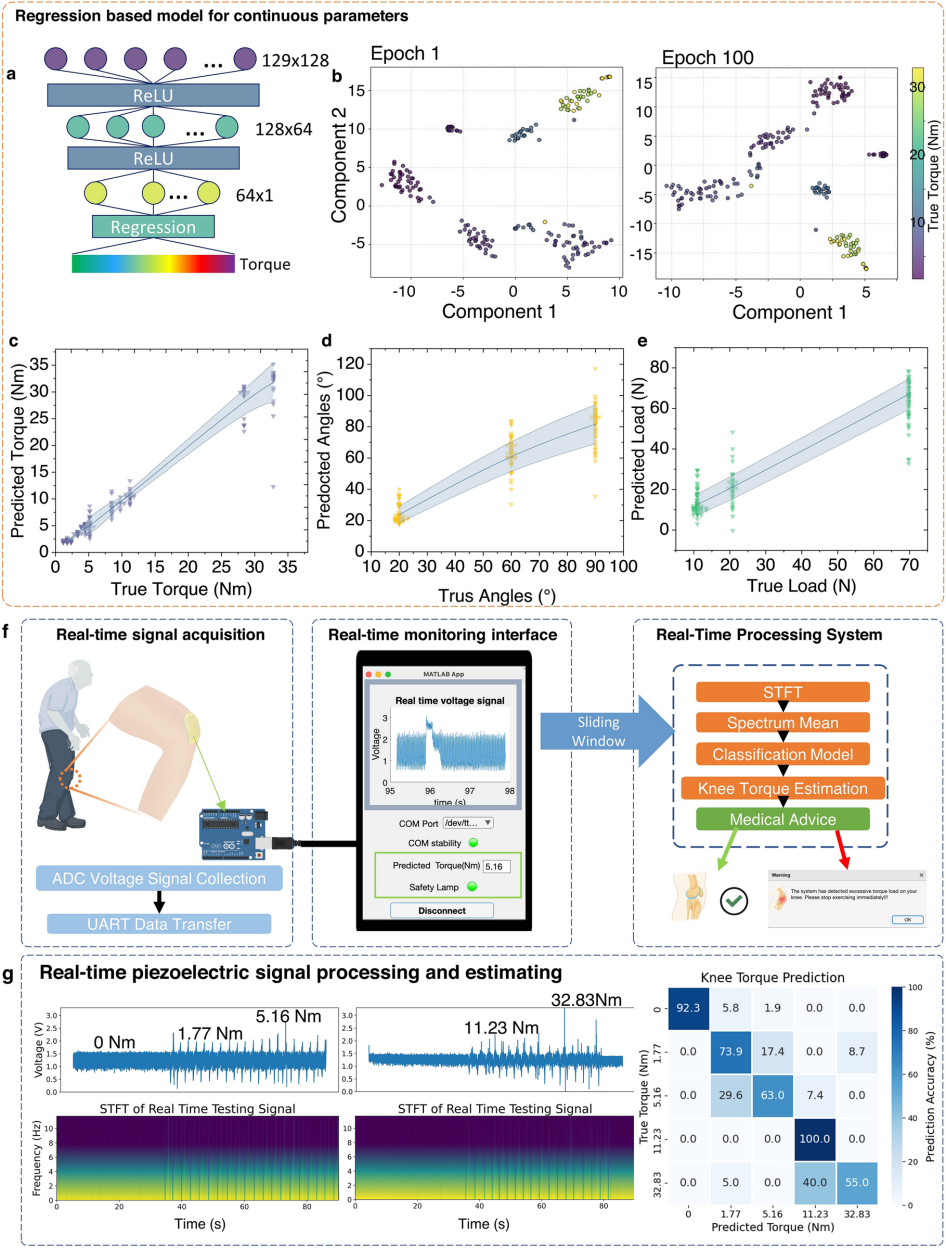
Regression based model and real-time knee torque estimation. **a** Structure of the classification model. **b** Feature vector matrix at epoch 1 and epoch 100 of the processing by the T-SNE algorithm. **c** Regression based prediction of knee joint torque; **d** prediction of knee bending angle and **e** prediction of knee loading. **f** Real-time estimation setups consisting real-time signal capturing, monitoring interfaces and real-time data processing. Voltage signals from the device were acquired using the ADC on Arduino board, digitized, and transmitted to a computer via UART for processing. The data is processed at real-time and estimated the torque to display on the interface. **g** 90 s example data of real-time capturing and professing and the accuracy of the real-time estimation of knee torque

With limited labels and datasets, the estimation of biomechanical parameters remains discrete, differing from the continuous and dynamically changing features observed in real-world scenarios. To address this limitation, we extended the classification model to a regression based approach, enabling the prediction of biomechanical features not explicitly included in the training data. This regression model allows for the estimation of continuous biomechanical parameters, even with a constrained training dataset, effectively bridging the gap between discrete data points and real-time dynamic variability. Figure 5a illustrates a regression based machine learning model designed to estimate continuous biomechanical parameters, including knee joint torque, bending angle, and load. The model architecture incorporates layered processing with ReLU-activated nodes, followed by a regression output layer, enabling continuous mapping of complex biomechanical relationships. Notably, the T-SNE visualization (Fig. 5b) reveals the progression of feature clustering during training. At epoch 1, the feature clusters appear more separated, reflecting the initial lack of correlation between groups of labelled signals. By epoch 100, the clusters become more interconnected, indicating the model's ability to establish relationships between signal features across groups, estimating more continuous and correlated features. The model demonstrates strong performance in predicting torque (Fig. 5c), bending angle (Fig. 5d), and load (Fig. 5e), with strong correlation between predicted and true values and minimal variance, as shown by the shaded confidence intervals. To assess the strength of the correlation, Pearson correlation coefficients were calculated for each model. The correlation coefficient for the torque prediction model was 0.9567, for the bending angle model was 0.9470, and for the knee angle model was 0.9118, indicating strong correlation between the predicted and actual values across all cases. An increase in variance was observed at higher torque levels, which can be attributed to two primary factors. First, higher torque movements introduce greater variability in motion execution, as participants experience muscle fatigue and involuntary micro-adjustments, leading to increased fluctuations in joint kinematics. This inconsistency in repeated movements contributes to a broader distribution of recorded signals, reducing prediction precision. Second, at higher torque values, soft tissue deformation and muscle co-contraction become more pronounced, leading to greater variations in sensor signals even for the same estimated torque. These biomechanical adaptations, including changes in muscle stiffness and antagonist muscle contributions, introduce inherent signal dispersion, increasing the uncertainty in torque estimation. Despite these challenges, the model maintains high accuracy across a wide range of motion conditions. This issue could be addressed by enhancing signal processing algorithms and adaptive calibration techniques to further optimize performance in high-torque scenarios. Unlike classification models, which provide discrete outputs, this regression based approach delivers continuous predictions, more closely describes the realistic dynamic biomechanical changes in torque, angle, and load. This continuous output is essential for applications requiring real-time adaptive monitoring, enabling a more comprehensive understanding of joint mechanics in natural environments.

We further extended the system's capability for real-time torque estimation. The nature of continuous real-time data is theoretically infinite in length and mainly dominated by resting-state signals. To enable real-time data processing, a dynamic sliding window approach was implemented. Considering the prevalence of resting states in real-time conditions, the training dataset for the model was augmented to additional data representing resting states (0 Nm), ensuring accurate prediction across all possible states. Figure 5f illustrates the real-time knee torque prediction system, which integrates signal acquisition, processing, and monitoring using a MATLAB application. Piezoelectric voltage signals, transmitted via UART, are displayed on the system's GUI in real-time. The system continuously evaluates torque predictions, triggering safety alerts, such as warning messages and a red lamp, estimating whether the torque exceeds predefined thresholds, thereby ensuring user safety. The real-time model predicts torque values and is displayed and stored along with raw data in the GUI. Figure 5g shows the system's example performance over a 160 s real-time session. The real-time system predicts five torque levels with an overall accuracy of 77.5%. The torque warning threshold was set at 30 Nm. Due to laboratory constraints and ethical considerations, potentially damaging torque levels were not introduced during the experiments. Instead, a conservative threshold of > 30 Nm was chosen to trigger warnings [46], as demonstrated in Video S1. This approach ensures user safety, while maintaining the effectiveness of the system in identifying high-torque scenarios. The predictions align closely with actual torque values, validating the model's accuracy and reliability for dynamic monitoring. This capability for continuous, real-time torque estimation makes the system a robust tool for applications in rehabilitation and injury prevention, while the safety features add value for practical, user-focused deployment. To evaluate the model’s adaptability to different users, a user-specific fine-tuning process was implemented. Each new participant followed a standardized movement collection protocol, generating a small personalized dataset for calibration. This fine-tuning approach allows the model to adjust to individual biomechanical variations, enhancing its robustness (Fig. S10a). In a validation test with an additional participant, the fine-tuned model achieved an accuracy of 78.8% (Fig. S10b), demonstrating its ability to generalize across different users.

## Discussion and Conclusions

In this study, we introduce an unconventional, piezoelectric boron nitride elastomer-based, AI-enabled wearable device specifically designed for continuous joint torque monitoring. Employing a reverse iterative design approach, we engineered wearable materials with an NPR (-0.94 with *a* = 10 mm, *b* = 3 mm and *θ* = 175°) finely tuned to match the biomechanical properties of the knee joint. This was coupled with the development of a highly sensitive piezoelectric film (with a sensitivity of 0.50 ± 0.01 V N^−1^), achieved by uniformly dispersing BNNTs into a PDMS matrix. The resulting film demonstrated the dual capabilities of accurately capturing knee motion and simultaneously enabling self-sufficient energy harvesting with a maximum power output of 1.47 mW m^−2^. To process the complex piezoelectric signals generated during movement, we integrated a lightweight, on-device ANN. This AI-driven system successfully extracted targeted signals from the intricate data output (with a high classification rate of 97.5%), subsequently mapping them to key physical parameters such as torque, angle, and loading. In the end, we constructed a real-time platform that demonstrated the robust capacity for real-time torque estimation using the wearable under dynamic conditions.

The proposed platform provides a non-invasive, indirect estimation of knee torque based on a relatively limited dataset collected from fundamental knee movements. As a proof-of-concept, the platform demonstrates promising accuracy in estimating knee dynamics, utilising a discrete knee torque training dataset and extending the mapping of movement signals to a continuous torque estimation model. While this approach serves as a valuable proof-of-concept, several limitations must be considered: (1) Dataset labelling and simplified torque modelling: The accuracy of knee torque labelling in this study is based on a simplified reference model, which is among the most basic mathematical models for estimating knee torque. The inherent limitation of this labelling approach stems from the difficulty of directly measuring knee torque in healthy individuals during dynamic movement. Currently, the only direct measurement method relies on instrumented knee prostheses, which are not applicable to intact biological joints. Therefore, the estimated torque values in this study may not fully capture the complexities of real-world knee biomechanics. Future research could explore alternative dataset collection methods, including direct torque measurements from cadaveric models or prosthetic knee implants, to improve estimation accuracy. (2) Extending from basic torque estimation to complex physiological states: The current study successfully estimates knee torque under controlled laboratory conditions using an indirectly measured dataset. In these fundamental movements, the estimated torque values align well with actual biomechanical expectations, representing the best achievable validation within a controlled experimental setting. However, applying this model to more complex motion states, such as walking, running, or jumping, presents greater challenges. While the model theoretically extends to these conditions, such estimations remain speculative, as current non-invasive techniques do not allow direct validation of knee torque during dynamic, high-intensity movements. Without direct measurement verification, conservative estimations were prioritized in this study rather than aggressive extrapolations. This can be addressed by incorporating cadaveric models or instrumented prosthetic joints to capture direct knee torque measurements under complex motion states, thereby refining the mapping between dynamic movement signals and knee joint mechanics.

In conclusion, this work presents a relatively low-cost and accessible solution for regular joint torque monitoring, making it suitable for populations across regions with varying levels of development. The proposed system has the potential to advance global efforts in joint health monitoring, the management of MSK conditions, rehabilitation, ageing disorders, and broader applications in personal healthcare. Future research will prioritize enhancing the system’s adaptability, scalability, and inclusivity. First, broadening its application to encompass various chronic conditions and diverse user groups, such as the elderly and individuals with MSK conditions and ageing disorders, will increase its practical clinical and healthcare applications in the field. Furthermore, investigating potential feasibilities to integrate extra complementary modalities, such as electricidal monitoring (e.g., EMG), and optical imaging (e.g., near-infrared spectroscopy), on the highly wearable flexible materials so as to offer unprecedented spatial–temporal resolution and patient comfort of joint torque monitoring. Besides this, augmenting the system’s resilience and ensuring seamless interoperability with external assistive technologies, including wearable robotics and exoskeletons, will substantially extend its functional scope and utility.

## Supplementary Information

Below is the link to the electronic supplementary material.

Supplementary file1 (DOCX 5127 KB)

Supplementary file2 (MP4 5655 KB)

## Data Availability

All code for this work is open accessed on GitHub. URL will be made available.

## References

[1] E. Sebbag, R. Felten, F. Sagez, J. Sibilia, H. Devilliers et al., The world-wide burden of musculoskeletal diseases: a systematic analysis of the world health organization burden of diseases database. Ann. Rheum. Dis. **78**(6), 844–848 (2019). 10.1136/annrheumdis-2019-215142 30987966 10.1136/annrheumdis-2019-215142

[2] A. Cieza, K. Causey, K. Kamenov, S.W. Hanson, S. Chatterji et al., Global estimates of the need for rehabilitation based on the global burden of disease study 2019: a systematic analysis for the global burden of disease study 2019. Lancet **396**(10267), 2006–2017 (2021). 10.1016/S0140-6736(20)32340-0 33275908 10.1016/S0140-6736(20)32340-0 PMC7811204

[3] M.D. National Academies of Sciences, Selected Health Conditions and Likelihood of Improvement with Treatment. *Selected Health Conditions and Likelihood of Improvement with Treatment* (National Academies Press, Washington, D.C., 2020). 32687289

[4] A. Braybrooke, K. Baraks, R. Burgess, A. Banerjee, J.C. Hill, Quality indicators for the primary and community care of musculoskeletal conditions: a systematic review. Arch. Phys. Med. Rehabil. **106**(3), 459–472 (2025). 10.1016/j.apmr.2024.08.022 39369932 10.1016/j.apmr.2024.08.022

[5] Musculoskeletal health. (2024). https://www.england.nhs.uk/elective-care-transformation/best-practice-solutions/musculoskeletal/

[6] H. Pang, S. Chen, D.M. Klyne, D. Harrich, W. Ding et al., Low back pain and osteoarthritis pain: a perspective of estrogen. Bone Res. **11**(1), 42 (2023). 10.1038/s41413-023-00280-x 37542028 10.1038/s41413-023-00280-x PMC10403578

[7] Versus Arthritis, The State of Musculoskeletal Health 2024. *The State of Musculoskeletal Health 2024* (Chesterfield, 2024).

[8] Office for Health Improvement and Disparities (OHID), Guidance Musculoskeletal health: applying All Our Health. (2022). https://www.gov.uk/government/publications/musculoskeletal-health-applying-all-our-health/musculoskeletal-health-applying-all-our-health

[9] J. Adamson, S. Ebrahim, P. Dieppe, K. Hunt, Prevalence and risk factors for joint pain among men and women in the West of Scotland twenty-07 study. Ann. Rheum. Dis. **65**(4), 520–524 (2006). 10.1136/ard.2005.037317 16126799 10.1136/ard.2005.037317 PMC1798081

[10] R.W. Nuckols, S. Lee, K. Swaminathan, D. Orzel, R.D. Howe et al., Individualization of exosuit assistance based on measured muscle dynamics during versatile walking. Sci. Robot. **6**(60), eabj1362 (2021). 10.1126/scirobotics.abj1362 34757803 10.1126/scirobotics.abj1362 PMC9052350

[11] Y. Jin, J.T. Alvarez, E.L. Suitor, K. Swaminathan, A. Chin et al., Estimation of joint torque in dynamic activities using wearable A-mode ultrasound. Nat. Commun. **15**(1), 5756 (2024). 10.1038/s41467-024-50038-0 38982087 10.1038/s41467-024-50038-0 PMC11233567

[12] A.E. Engin, M.S. Korde, Biomechanics of normal and abnormal knee joint. J. Biomech. **7**(4), 325–334 (1974). 10.1016/0021-9290(74)90027-X 4412204 10.1016/0021-9290(74)90027-x

[13] E.G. Meyer, R.C. Haut, Excessive compression of the human tibio-femoral joint causes ACL rupture. J. Biomech. **38**, 2311–2316 (2005). 10.1016/j.jbiomech.2004.10.003 16154419 10.1016/j.jbiomech.2004.10.003

[14] J. Verheul, N.J. Nedergaard, J. Vanrenterghem, M.A. Robinson, Measuring biomechanical loads in team sports–from lab to field. Sci. Med. Footb. **4**(3), 246–252 (2020). 10.1080/24733938.2019.1709654

[15] D.S. Logerstedt, J.R. Ebert, T.D. MacLeod, B.C. Heiderscheit, T.J. Gabbett et al., Effects of and response to mechanical loading on the knee. Phys. Med. **52**(2), 201–235 (2022). 10.1007/s40279-021-01579-7 10.1007/s40279-021-01579-7 34669175

[16] B. Innocenti, Biomechanics of the knee joint, in *Human Orthopaedic Biomechanics*. (Elsevier, Netherland, 2022), pp.239–263

[17] L. Zhang, G. Liu, B. Han, Z. Wang, Y. Yan et al., Knee joint biomechanics in physiological conditions and how pathologies can affect it: a systematic review. Appl. Bionics Biomech. **2020**, 7451683 (2020). 10.1155/2020/7451683 32322301 10.1155/2020/7451683 PMC7160724

[18] S.E. Forrester, M.R. Yeadon, M.A. King, M.G. Pain, Comparing different approaches for determining joint torque parameters from isovelocity dynamometer measurements. J. Biomech. **44**(5), 955–961 (2011). 10.1016/j.jbiomech.2010.11.024 21159340 10.1016/j.jbiomech.2010.11.024

[19] J. Holder, U. Trinler, A. Meurer, F. Stief, A systematic review of the associations between inverse dynamics and musculoskeletal modeling to investigate joint loading in a clinical environment. Front. Bioeng. Biotechnol. **8**, 603907 (2020). 10.3389/fbioe.2020.603907 33365306 10.3389/fbioe.2020.603907 PMC7750503

[20] M. Safaei, R. Michael Meneghini, S.R. Anton, Force detection, center of pressure tracking, and energy harvesting from a piezoelectric knee implant. Smart Mater. Struct. **27**(11), 114007 (2018). 10.1088/1361-665X/aad755 30297976 10.1088/1361-665X/aad755 PMC6173487

[21] C. Jacq, T. Maeder, S. Emery, M. Simoncini, E. Meurville et al., Investigation of polymer thick-film piezoresistors for medical wrist rehabilitation and artificial knee load sensors. Procedia Eng. **87**, 1194–1197 (2014). 10.1016/j.proeng.2014.11.380

[22] M. Arvanitidis, D. Jiménez-Grande, N. Haouidji-Javaux, D. Falla, E. Martinez-Valdes, People with chronic low back pain display spatial alterations in high-density surface EMG-torque oscillations. Sci. Rep. **12**(1), 15178 (2022). 10.1038/s41598-022-19516-7 36071134 10.1038/s41598-022-19516-7 PMC9452584

[23] S. Yang, J. Cheng, J. Shang, C. Hang, J. Qi et al., Stretchable surface electromyography electrode array patch for tendon location and muscle injury prevention. Nat. Commun. **14**(1), 6494 (2023). 10.1038/s41467-023-42149-x 37838683 10.1038/s41467-023-42149-x PMC10576757

[24] J. Chapman, A. Dwivedi, M. Liarokapis, A wearable, open-source, lightweight forcemyography armband: on intuitive, robust muscle-machine interfaces. In 2021 IEEE/RSJ International Conference on Intelligent Robots and Systems (IROS). September 27-October 1, 2021. Prague, Czech Republic. IEEE, (2021). pp. 4138–4143. 10.1109/iros51168.2021.9636345

[25] Z.G. Xiao, C. Menon, Performance of forearm FMG and sEMG for estimating elbow, forearm and wrist positions. J. Bionic Eng. **14**(2), 284–295 (2017). 10.1016/S1672-6529(16)60398-0

[26] Z. Qing, Z. Lu, Z. Liu, Y. Cai, S. Cai et al., A simultaneous gesture classification and force estimation strategy based on wearable A-mode ultrasound and cascade model. IEEE Trans. Neural Syst. Rehabil. Eng. **30**, 2301–2311 (2022). 10.1109/TNSRE.2022.3196926 35930512 10.1109/TNSRE.2022.3196926

[27] D. Kim, J. Ko, Y.-K. Kim, S.S. Lee, S. Ahn et al., Spontaneous alignment of boron nitride nanotubes into polycrystalline film arrays for enhanced piezoelectric nanogeneration. Small Struct. **5**(11), 2400259 (2024). 10.1002/sstr.202400259

[28] A. Niguès, A. Siria, P. Vincent, P. Poncharal, L. Bocquet, Ultrahigh interlayer friction in multiwalled boron nitride nanotubes. Nat. Mater. **13**(7), 688–693 (2014). 10.1038/nmat3985 24880730 10.1038/nmat3985

[29] Y. Huang, J. Lin, J. Zou, M.-S. Wang, K. Faerstein et al., Thin boron nitride nanotubes with exceptionally high strength and toughness. Nanoscale **5**(11), 4840–4846 (2013). 10.1039/c3nr00651d 23615971 10.1039/c3nr00651d

[30] X. Zeng, J. Sun, Y. Yao, R. Sun, J.-B. Xu et al., A combination of boron nitride nanotubes and cellulose nanofibers for the preparation of a nanocomposite with high thermal conductivity. ACS Nano **11**(5), 5167–5178 (2017). 10.1021/acsnano.7b02359 28402626 10.1021/acsnano.7b02359

[31] J.H. Kang, G. Sauti, C. Park, V.I. Yamakov, K.E. Wise et al., Multifunctional electroactive nanocomposites based on piezoelectric boron nitride nanotubes. ACS Nano **9**(12), 11942–11950 (2015). 10.1021/acsnano.5b04526 26529472 10.1021/acsnano.5b04526

[32] J. Zhang, S. Ye, H. Liu, X. Chen, X. Chen et al., 3D printed piezoelectric BNNTs nanocomposites with tunable interface and microarchitectures for self-powered conformal sensors. Nano Energy **77**, 105300 (2020). 10.1016/j.nanoen.2020.105300

[33] P. Snapp, C. Cho, D. Lee, M.F. Haque, S. Nam et al., Tunable piezoelectricity of multifunctional boron nitride nanotube/poly(dimethylsiloxane) stretchable composites. Adv. Mater. **32**, 2004607 (2020). 10.1002/adma.202004607 10.1002/adma.202004607 32954543

[34] B.J. Stetter, T. Stein, Machine learning in biomechanics: enhancing human movement analysis, in *Artificial Intelligence in Sports, Movement, and Health*. (Springer Nature Switzerland, Cham, 2024), pp.139–160

[35] Y. Guo, H. Zhang, L. Fang, Z. Wang, W. He et al., A self-powered flexible piezoelectric sensor patch for deep learning-assisted motion identification and rehabilitation training system. Nano Energy **123**, 109427 (2024). 10.1016/j.nanoen.2024.109427

[36] Y. Chen, X. Zhang, C. Lu, Flexible piezoelectric materials and strain sensors for wearable electronics and artificial intelligence applications. Chem. Sci. **15**(40), 16436–16466 (2024). 10.1039/d4sc05166a 39355228 10.1039/d4sc05166a PMC11440360

[37] Y. Luo, Y. Li, P. Sharma, W. Shou, K. Wu et al., Learning human–environment interactions using conformal tactile textiles. Nat. Electron. **4**(3), 193–201 (2021). 10.1038/s41928-021-00558-0

[38] U.D. Larsen, O. Signund, S. Bouwsta, Design and fabrication of compliant micromechanisms and structures with negative Poisson’s ratio. J. Microelectromech. Syst. **6**(2), 99–106 (1997). 10.1109/84.585787

[39] L. Mizzi, E. Salvati, A. Spaggiari, J.-C. Tan, A.M. Korsunsky, 2D auxetic metamaterials with tuneable micro-/nanoscale apertures. Appl. Mater. Today **20**, 100780 (2020). 10.1016/j.apmt.2020.100780

[40] L. Mizzi, E. Salvati, A. Spaggiari, J.-C. Tan, A.M. Korsunsky, Highly stretchable two-dimensional auxetic metamaterial sheets fabricated *via* direct-laser cutting. Int. J. Mech. Sci. **167**, 105242 (2020). 10.1016/j.ijmecsci.2019.105242

[41] J.C. Lagarias, J.A. Reeds, M.H. Wright, P.E. Wright, Convergence properties of the nelder: mead simplex method in low dimensions. SIAM J. Optim. **9**(1), 112–147 (1998). 10.1137/s1052623496303470

[42] A.F. Möslein, M. Gutiérrez, B. Cohen, J.-C. Tan, Near-field infrared nanospectroscopy reveals guest confinement in metal-organic framework single crystals. Nano Lett. **20**(10), 7446–7454 (2020). 10.1021/acs.nanolett.0c02839 32870694 10.1021/acs.nanolett.0c02839

[43] D. Griffin, J. Lim, Signal estimation from modified short-time Fourier transform. IEEE Trans. Acoust. Speech Signal Process. **32**(2), 236–243 (1984). 10.1109/TASSP.1984.1164317

[44] A.C. Belkina, C.O. Ciccolella, R. Anno, R. Halpert, J. Spidlen et al., Automated optimized parameters for T-distributed stochastic neighbor embedding improve visualization and analysis of large datasets. Nat. Commun. **10**(1), 5415 (2019). 10.1038/s41467-019-13055-y 31780669 10.1038/s41467-019-13055-y PMC6882880

[45] Dina Zhabinskaya, University of California Davis: Physics 7B General Physics. *University of California Davis: Physics 7B General Physics* (University of California Davis, n.d.).

[46] R. Riemer, E.T. Hsiao-Wecksler, Improving joint torque calculations: optimization-based inverse dynamics to reduce the effect of motion errors. J. Biomech. **41**(7), 1503–1509 (2008). 10.1016/j.jbiomech.2008.02.011 18396292 10.1016/j.jbiomech.2008.02.011

