# Supplementary material for: AI-Enabled Piezoelectric Wearable for Joint Torque Monitoring
Source: Nanomicro Lett. 2025 May 3;17:247. doi: 10.1007/s40820-025-01753-w (PMC12048387; doi:10.1007/s40820-025-01753-w)
Supplement: Supplementary file 1 — Supplementary file1 (DOCX 5127 KB) [file 40820_2025_1753_MOESM1_ESM.docx]

Supporting Information for

**AI-Enabled Piezoelectric Wearable for Joint Torque Monitoring**

Jinke Chang^1, 2^, Jinchen Li^2^, Jiahao Ye^1^, Bowen Zhang^2,3^, Jianan Chen^2^, Yunjia Xia^2^, Jingyu Lei^2^, Tom Carlson^2^, Rui Loureiro^2^, Alexander M. Korsunsky^4^, Jin-Chong Tan^1,^* and Hubin Zhao^2,^ *

^1^Multifunctional Materials & Composites (MMC) Laboratory, Department of Engineering Science, University of Oxford, Oxford OX1 3PJ, UK

^2^HUB of Intelligent Neuro-engineering (HUBIN), Aspire CREATe, DSIS, University College London, London HA7 4LP, UK

^3^School of Mechanical Engineering, State Key Laboratory for Manufacturing System Engineering, Xi'an Jiaotong University, Xi’an 710054, P. R. China

^4^Trinity College, University of Oxford, Oxford OX1 3BH, UK

*Corresponding authors. E-mail: [jin-chong.tan@eng.ox.ac.uk](mailto:jin-chong.tan@eng.ox.ac.uk) (Jin-Chong Tan); [hubin.zhao@ucl.ac.uk](mailto:hubin.zhao@ucl.ac.uk) (Hubin Zhao)

**Supplementary Tables and Figures**

**Table S1** Comparison of indirect and direct measurement of knee dynamics

|  | Method | Material | Advantage | Disadvantage | Refs. |
| --- | --- | --- | --- | --- | --- |
| Indirect | Sleeve-type wearable sensor measurements | PVDF/BT10 | Non-invasive, flexible, self-powered | Complex data processing, environmental sensitivity, calibration issues | [47] |
|  | Patch-type wearable sensor measurements | PLLA/glycine | Non-invasive, self-powered, biodegradable, flexible and lightweight, high sensitivity | Limited signal strength, potential noisy sensitivity, degradability concerns. | [48] |
|  | Patch-type wearable sensor | BaTiO₃ | Non-invasive, skin-conformal and flexible, self-powered, high sensitivity, durability | Limited to motion detection, potential sensitivity to external factors | [49] |
|  | Dynamometer-based measurement | - | Non-invasive, applicable for clinical and research use | Limited to laboratory settings, expensive and bulky | [18] |
|  | Inverse dynamics computational modeling | - | Non-invasive, applicable to dynamic movements, commonly used in research | Sensitive to errors, assume rigid body dynamics, limited accuracy in real-world conditions | [19] |
| Direct | Piezoelectric sensor embedded in knee prosthesis | PZT-5A | Direct measurement of knee joint force, self-powered, real-time force sensing, tracks center of pressure | Invasive, implant design modification required, potential material fatigue, limited to post-surgical patients | [20] |
|  | Piezoresistive sensor embedded in knee prothesis | FR4 substrate/ piezoresistive ink | High sensitivity, compact and flexible, real-time force monitoring | Invasive, signal drift, calibration issues, limited to total knee arthroplasty patients | [21] |
|  | Strain gauges embedded in total knee arthroplasty | - | Long-term monitoring, validation of computational models | Invasive, signal drift over long periods, power dependency, restricted to total knee arthroplasty patients | [50] |
|  | Magnetoresistive sensor embedded in joint implant | Sm_2_Co_17_/NdFeB/InSb | Minimally invasive monitoring | Invasive, temperature sensitivity, calibration issues, not suitable for long term, external power dependence | [51] |
|  | Magnetoresistive sensor embedded in joint implant | Sm_2_Co_17_/InSb | Self-powered, compact | Invasive, limited accuracy, temperature sensitivity, complex implantation | [52] |


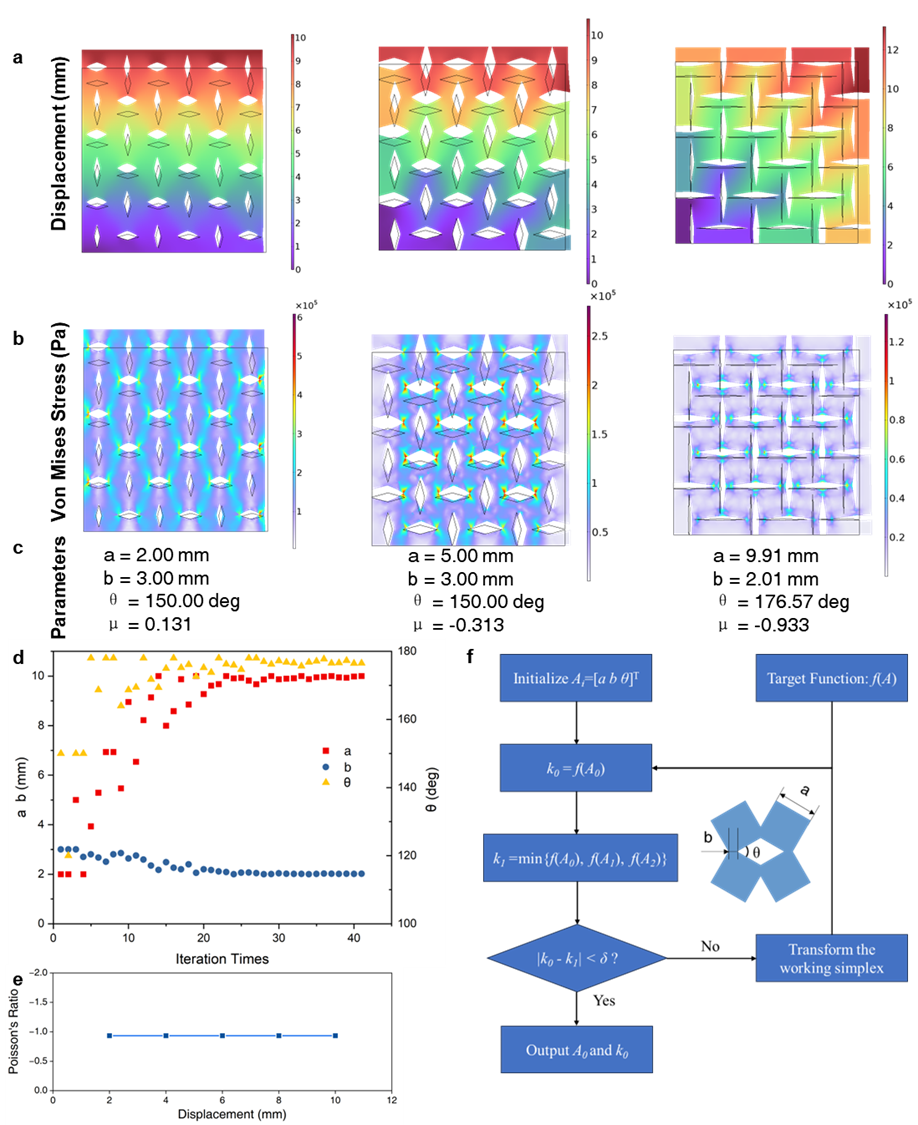


**Fig. S1** Finite element analysis for the inverse design of a Poisson’s ratio-matched knee monitoring structure. **a** Displacement and **b** Von Mises stress distributions for three parameter optimization examples targeting a specific Poisson’s ratio. **c** Parameter optimization process over 40 iterations to achieve the targeted Poisson’s ratio. **d** Optimized Poisson’s ratio and corresponding relative displacement. **e** Calculated Poisson’s ratio vs displacement. **f** Nelder Mead algorithm workflow for structure optimisation, where the target function has been defined in Eq. (5) and *δ* was set as 0.01

The Poisson’s ratio of the whole structure of fabricated patch was measured and calculated. The length and width of the patch without loading were measured by a vernier calliper noted as $L_{1}$ and $W_{1}$, respectively. Then one side of the patch was fixed by tape, and the other side was stretched by 10 mm along the length direction, leading to a deformation of the width of the patch measured as $W_{2}$. The Poisson’s Ratio of the whole patch has been calculated using Eq. (6). The Poisson's ratio of the simulated periodic structure should match that of the actual fabricated patch. To ensure that, the length and width of the patch were integer multiples of the unit structure period during fabrication, avoiding Poisson's ratio bias caused by non-integer periodic structures.

**Table S2** Design of wearable devices for joint monitoring

| Device Type | Wearable Knee Joint Monitoring Device | Description | Design Features | References |
| --- | --- | --- | --- | --- |
| Hinged | 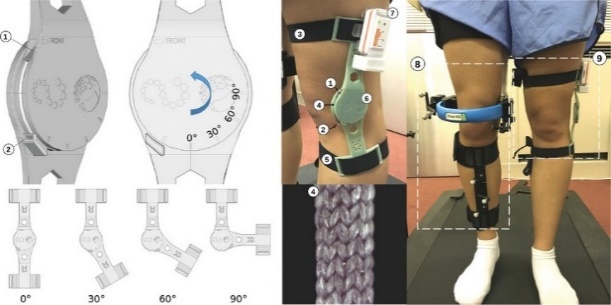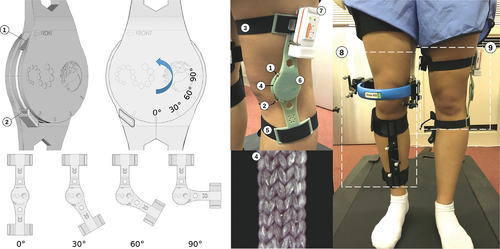 | Knee brace with textile yarns as sensor embedded inside the central circular section | ① Holders near the knee joint with hinge that adapts to the bending motion of the knee  ② Suitable for sensors measuring joint rotation | Reproduced from [53], with permission from © 2022 The Authors. Advanced Sensor Research, published by Wiley‐VCH GmbH |
|  | 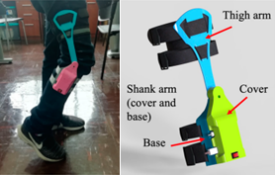 | A knee wearable with sensors embedded inside the shank arm structure |  | Reproduced from [54] Copyright @2022 MDPI, under CC BY license. |
|  | 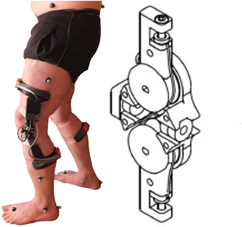 | Tensile “ligament” structure for the cruciate hinge with a rotary position sensor in each pivot point |  | Reproduced from [55] with permission @ 2021 IPEM. Published by Elsevier Ltd. |
| Strap | 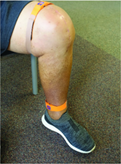 | Ankle-worn and thigh-worn inertial measurement units | ① Sensors with straps attached on the thigh or ankle  ② Suitable for three-dimensional electronic measurement units | Reproduced from [56] Copyright @2021 MDPI, under CC BY license |
|  | 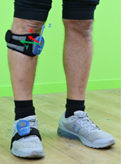 | Wearable magneto-inertial measurement units were attached on participants using ad hoc straps |  | Reproduced from [57] Copyright: © 2022 Baldazzi et al. under CC AL license |
|  | 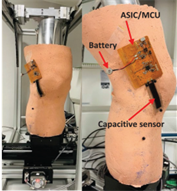 | Wearable capacitive-based sensor system that can be worn as a patch |  | Reproduced from [58] Copyright@ 2023 The Authors. Advanced Sensor Research published by Wiley‐VCH GmbH, under CCBY license |


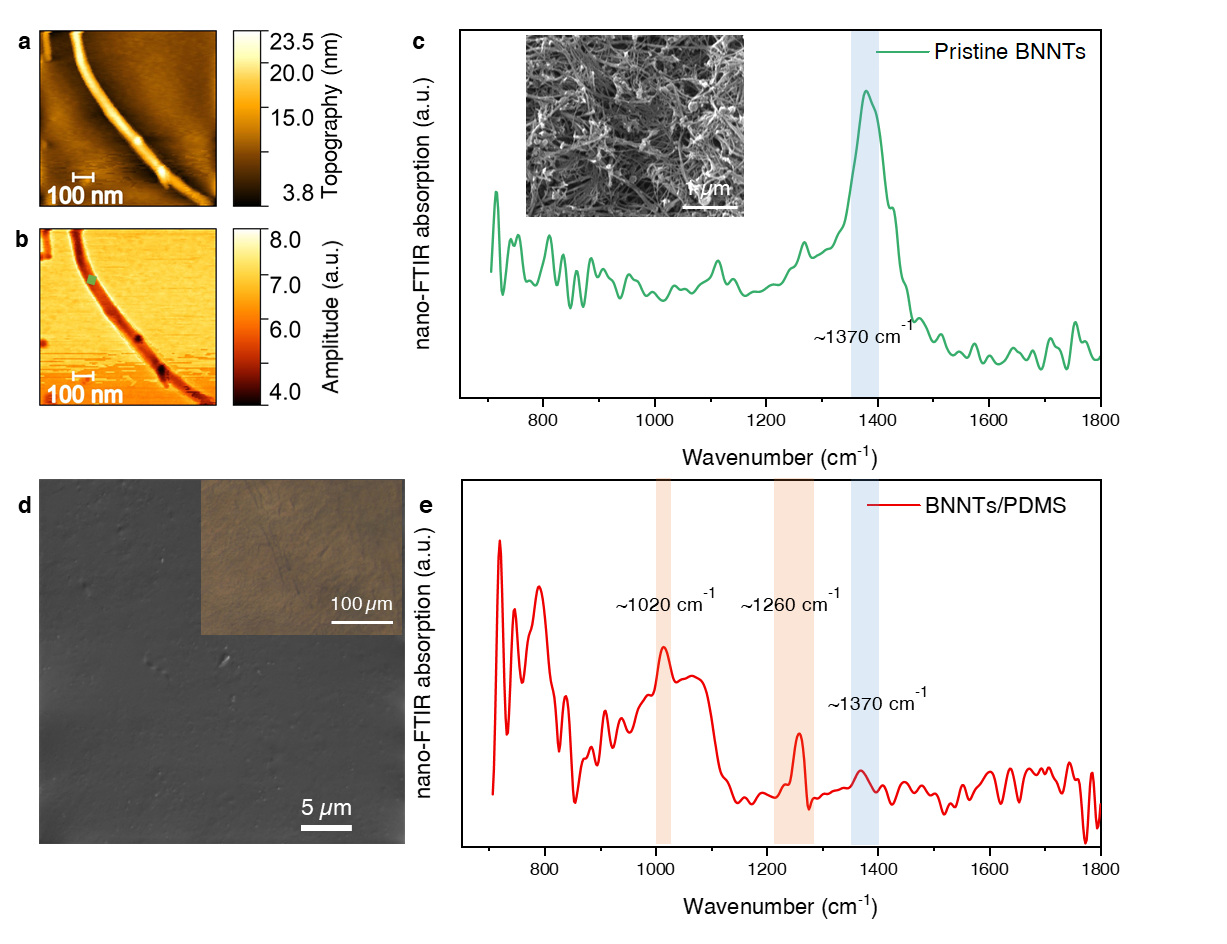


**Fig. S2** Characterization of BNNTs and BNNTs/PDMS composite. **a** AFM topography image. **b** s-SNOM amplitude mapping of pristine BNNTs dispersed on a silicon wafer. **c** Nano-FTIR absorption spectrum of a single nanotube, taken from the green point indicated in **b**. **d** SEM image of the BNNTs/PDMS surface, showing a smooth and uniform distribution of the composite material. Inset: optical stereo transmission imaging of the composite material. **e** Nano-FTIR absorption spectrum of the BNNTs/PDMS surface, taken from a point with a small BNNT cluster intentionally selected for analysis. The surface was otherwise uniform, with minimal nano-FTIR responses from BNNTs due to the technique’s spatial resolution of <20 nm.
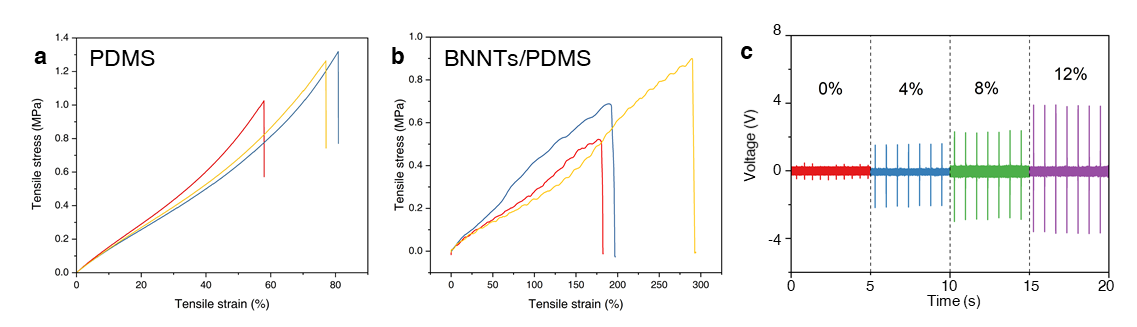


**Fig. S3** Mechanical and electrical properties of PDMS and BNNTs/PDMS composite. **a** Strain-stress curves of three representative PDMS samples. **b** Strain-stress curves of three representative BNNTs/PDMS composite samples. **c** Voltage output variation with different BNNT/PDMS composite ratios

*
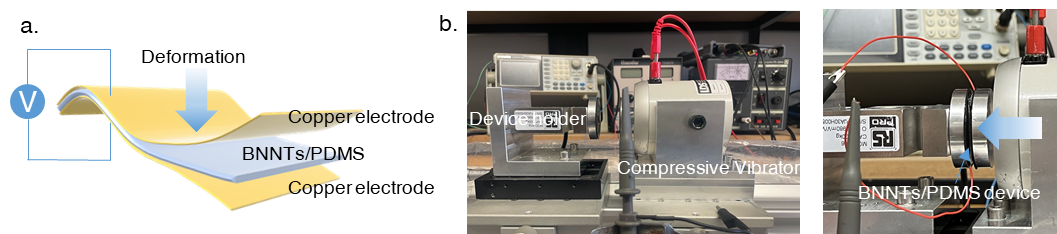
*

**Fig. S4** Device and setup for standard electrical performance testing of BNNTs/PDMS composite. **a** Illustration of the device used in the standard piezoelectric performance test, consisting of a 2 × 2 cm composite material sandwiched between two copper electrodes, with the electrical output measured from the electrodes. **b** Photograph of the electrical performance testing setup, showing the vibrator compressing the device, which is securely fixed on a sample holder on the left

**Table S3** Summary of piezoelectric performance of film materials associated with BNNTs and PDMS

| Material | Area (cm*cm) | Voltage (V) | Current | Sensitivity | | Refs. |
| --- | --- | --- | --- | --- | --- | --- |
| BNNT/PDMS | 2.0 × 2.0 | 5.76 ± 0.02 | 6.9 ± 0.42 mA/m² | | 0.5 ± 0.007 V/N | This work |
| Cellulose/PLLA | 1.8 × 2.8 | 10.3 | 261.8 nA | | 0.57 V/N | [59] |
| BNNT/PVDF | 3.0 × 4.0 | 0.128 ± 0.0054 | N/A | | ~0.4 mV/kPa | [60] |
| HFP/ PVDF | 1.0 × 1.0 | 4.6 | N/A | | 0.117 V/N | [61] |
| ZnO/AlN/PDMS | 3.5 × 2.0 | 3.631 | N/A | | 8.77 mV/kPa | [62] |
| BaTiO3/PVDF | 1.8 × 2.0 | 9.3 | 86 nA | | ~0.056 V/N | [63] |
| ZnO/PVDF | 3.0 × 3.0 | ~3 | 254 nA | | 0.37 mV/kPa | [64] |
| BNNS/PDMS | 4.5 × 2.5 | 22 | 75 nA | | ~0.048 V/N | [65] |
| PZT/PDMS | 2.0 × 2.0 | 2.34 | N/A | | 8.59 mV/kPa | [66] |
| BNNT/PDMS | 1.0 × 0.5 | 0.4 | N/A | | N/A | [67] |
| BNNT-ZnO QDs/PDMS | 3.0 × 3.0 | 18.56 | 734.5 nA | | N/A | [68] |
| F-BNNTs/Resin | 1.0 × 1.0 | 9.6 | N/A | | 24 mV/kPa | [69] |
| PLA/BTO | 11 × 11 | 20.17 | 261.8 nA | | 176 mV/kPa | [70] |


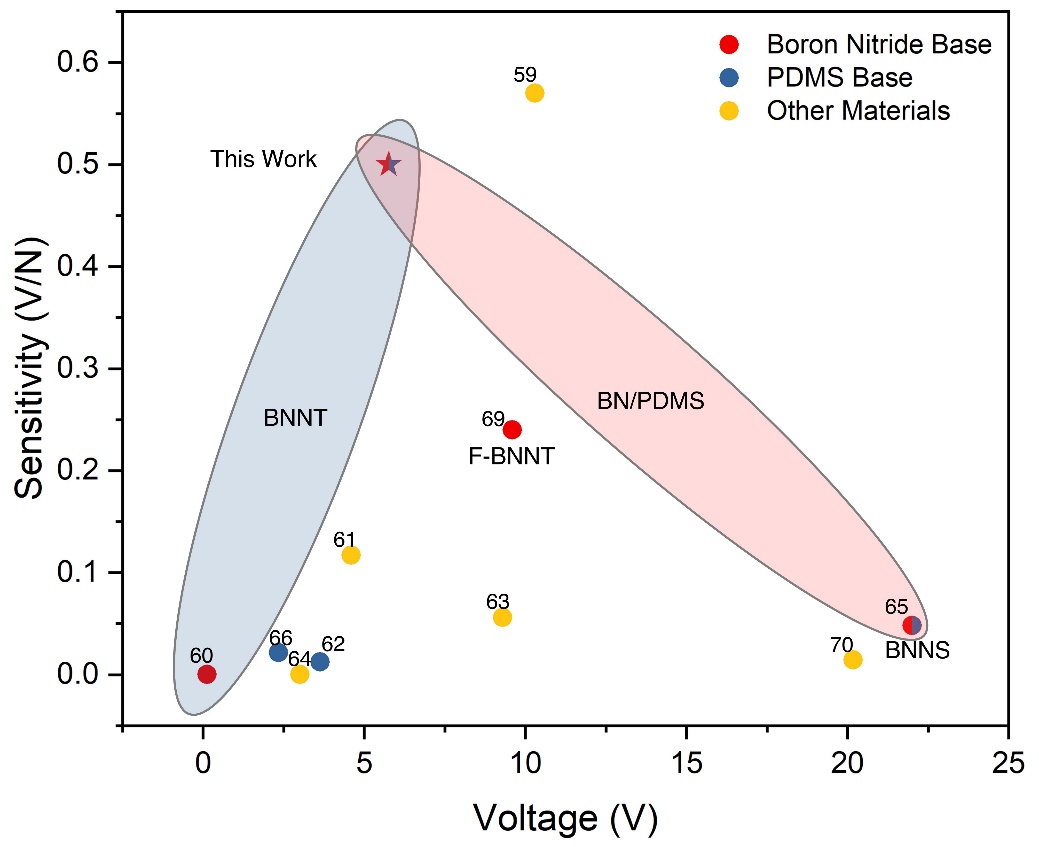


**Fig. S5** Comparison of the piezoelectric performance of boron nitride-based and PDMS-based materials


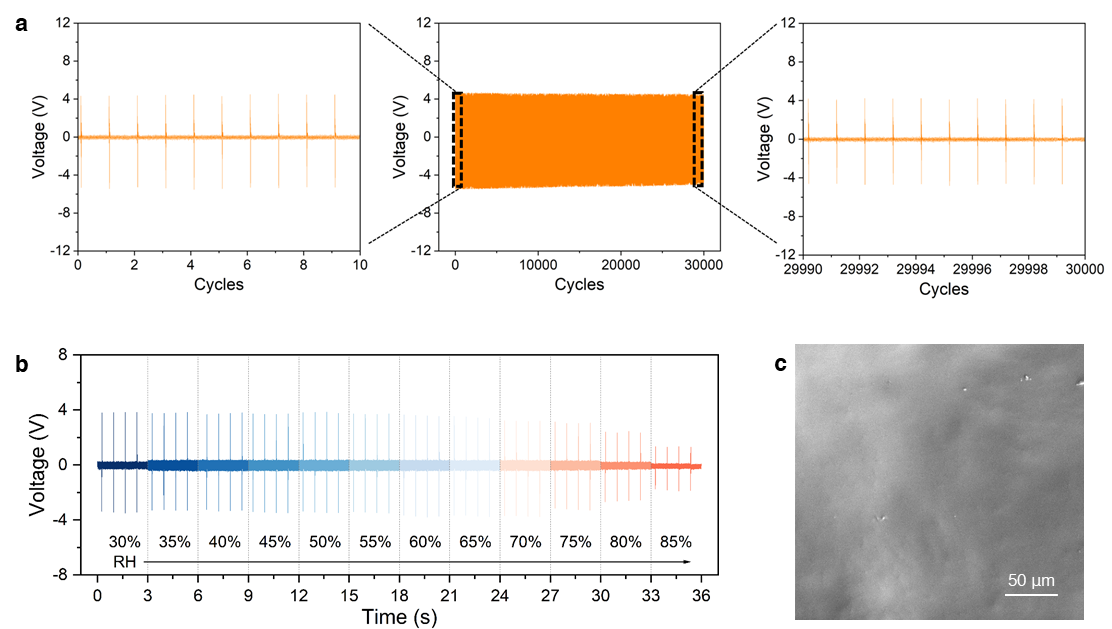


**Fig. S6** Device durability tests. **a** Long-term durability of BNNTs/PDMS device over a continuous running test of 30,000 cycles. **b** Voltage output of the device under different relative humidity. **c** SEM image of BNNTs/PDMS sample surface after durability test


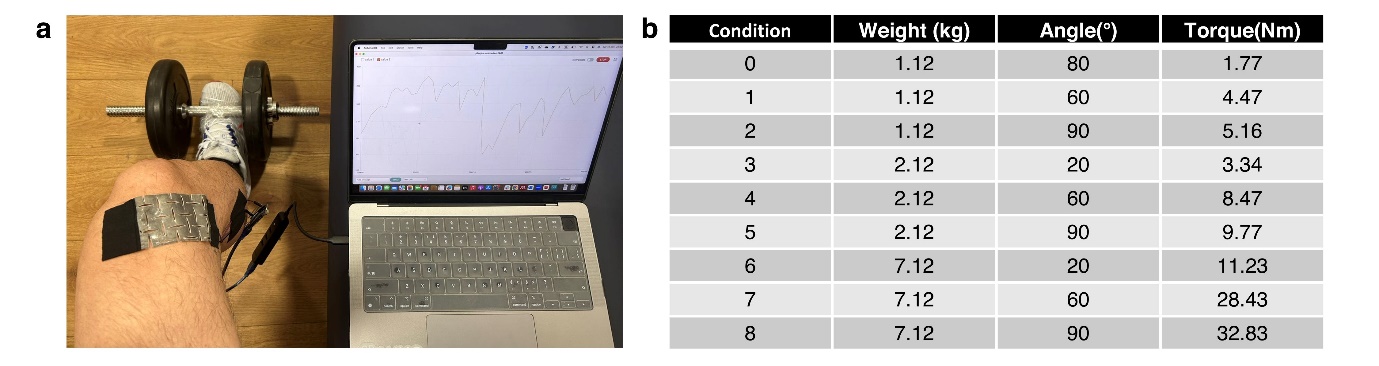


**Fig. S7** Training database collection for machine learning. **a** Example photograph showing the collection of knee joint motion signals from a participant. **b** Experimental conditions and labels indicating the physical significance of the training datasets


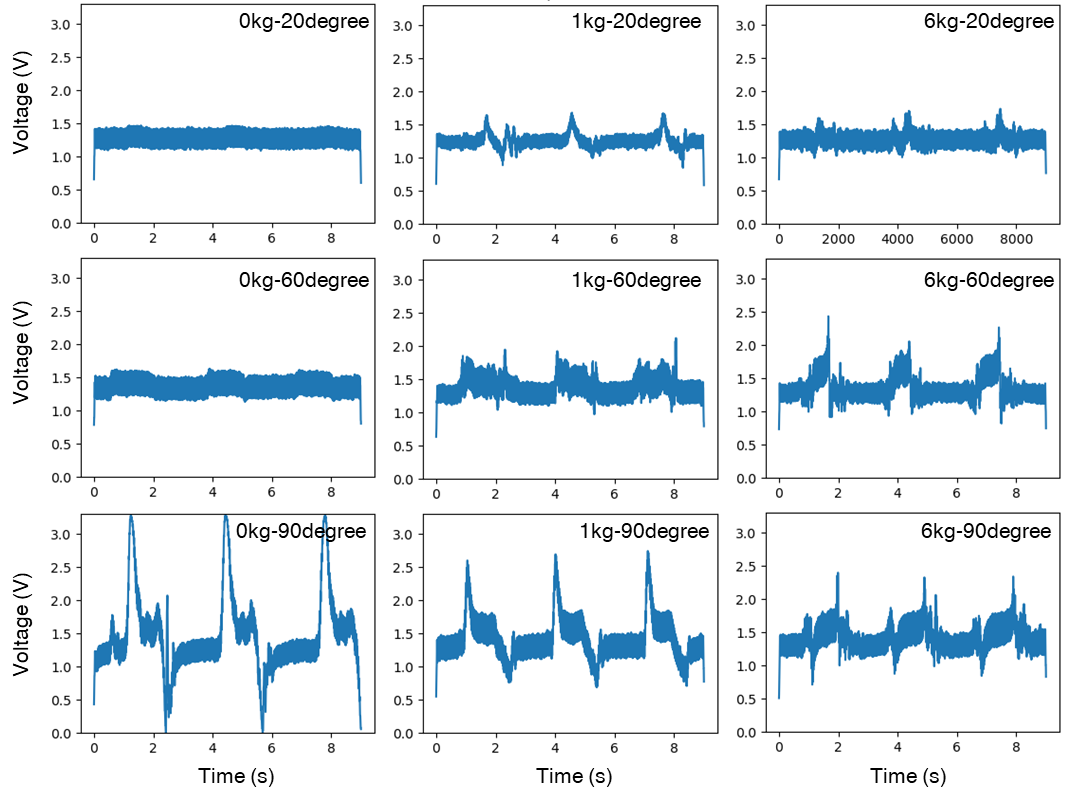


**Fig. S8** Typical voltage responses of the BNNTs/PDMS device under different motions and loads. The voltage output was recorded for various loading weights (0, 1, and 6 kg) and knee bending angles (20°, 60°, and 90°)


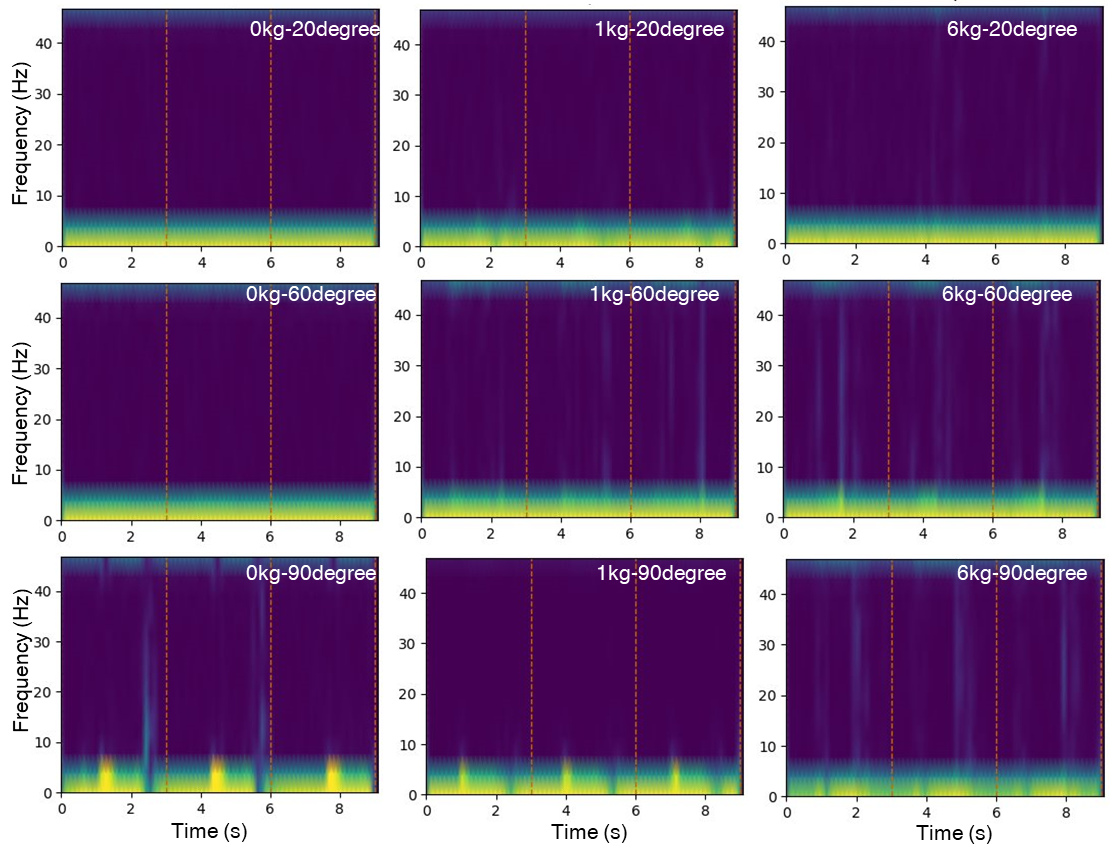


**Fig. S9** STFT spectrum of typical voltage responses of the BNNTs/PDMS device under different motions and loads


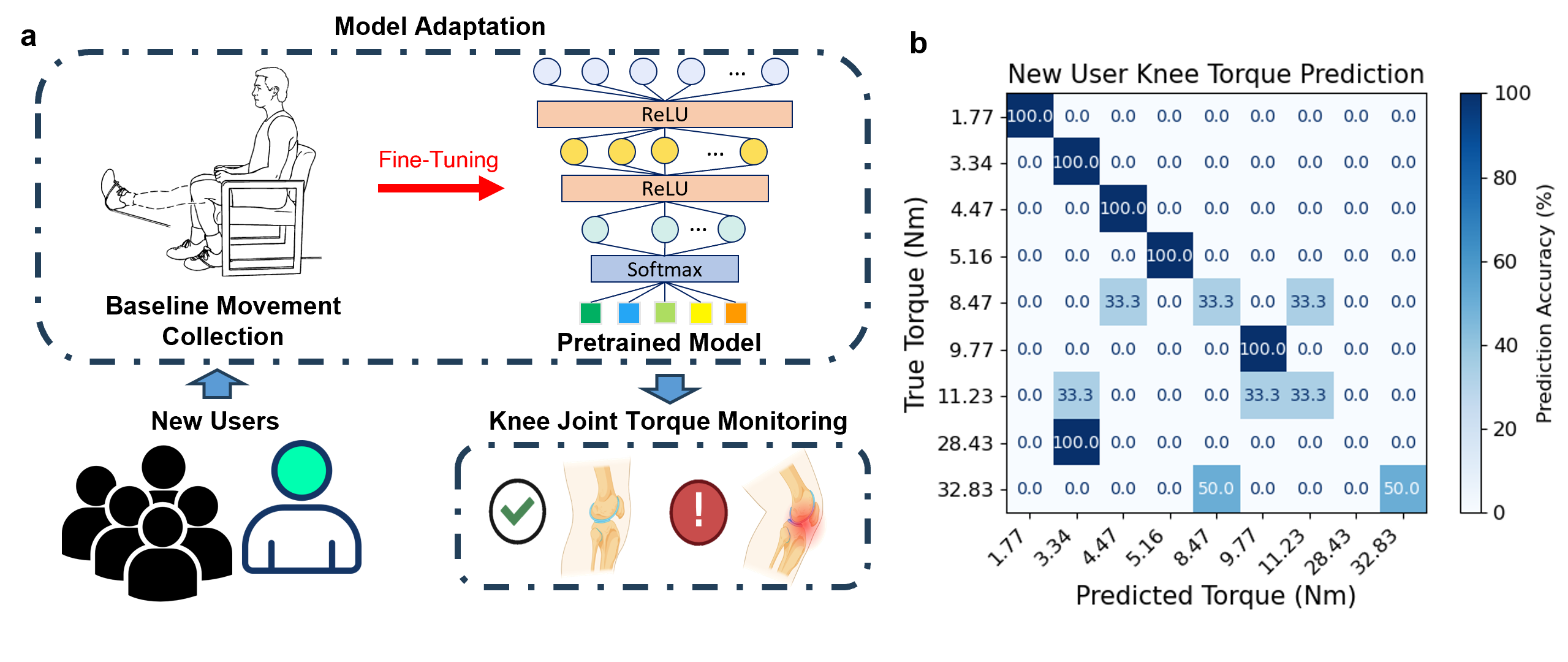


**Fig. S10** **a** Workflow of new users’ adaption and personalized fine tuning. **b** Confusion matrix of the fine-tuned torque prediction model

**Supporting Video S1**: Real-time estimation of knee torque

**References**

[47] F. Mokhtari, G.M. Spinks, C. Fay, Z. Cheng, R. Raad et al., Wearable electronic textiles from nanostructured piezoelectric fibers. Adv. Mater. Technol. **5**(4), 1900900 (2020). <https://doi.org/10.1002/admt.201900900>

[48] M. Ali, S.M. Hoseyni, R. Das, M. Awais, I. Basdogan et al., A flexible and biodegradable piezoelectric-based wearable sensor for non-invasive monitoring of dynamic human motions and physiological signals. Adv. Mater. Technol. **8**(15), 2300347 (2023). <https://doi.org/10.1002/admt.202300347>

[49] J. Yu, X. Hou, M. Cui, N. Zhang, S. Zhang et al., Skin-conformal BaTiO_3_/ecoflex-based piezoelectric nanogenerator for self-powered human motion monitoring. Mater. Lett. **269**, 127686 (2020). <https://doi.org/10.1016/j.matlet.2020.127686>

[50] D.D. D’Lima, B.J. Fregly, C.W. Colwell Jr, Implantable sensor technology: measuring bone and joint biomechanics of daily life *in vivo*. Arthritis Res. Ther. **15**(1), 203 (2013). <https://doi.org/10.1186/ar4138>

[51] D. Crescini, E. Sardini, M. Serpelloni, An autonomous sensor for force measurements in human knee implants. Procedia Chem. **1**(1), 718–721 (2009). <https://doi.org/10.1016/j.proche.2009.07.179>

[52] M.A. Khan, M. Borghetti, M. Serpelloni, E. Sardini, Implantable autonomous device for wireless force measurement in total knee prosthesis. IEEE Instrum. Meas. Mag. **22**(1), 39–47 (2019). <https://doi.org/10.1109/MIM.2019.8633351>

[53] C.D. Fay, N. Mannering, A. Jeiranikhameneh, F. Mokhtari, J. Foroughi et al., Wearable carbon nanotube-*Spandex* textile yarns for knee flexion monitoring. Adv. Sens. Res. **2**(1), 2200021 (2023). <https://doi.org/10.1002/adsr.202200021>

[54] B. Rivera, C. Cano, I. Luis, D.A. Elias, A 3D-printed knee wearable goniometer with a mobile-app interface for measuring range of motion and monitoring activities. Sensors **22**(3), 763 (2022). <https://doi.org/10.3390/s22030763>

[55] C. Young, M.L. Oliver, K.D. Gordon, Design and validation of a novel 3D-printed wearable device for monitoring knee joint kinematics. Med. Eng. Phys. **94**, 1–7 (2021). <https://doi.org/10.1016/j.medengphy.2021.05.013>

[56] S.M. Bolam, B. Batinica, T.C. Yeung, S. Weaver, A. Cantamessa et al., Remote patient monitoring with wearable sensors following knee arthroplasty. Sensors **21**(15), 5143 (2021). <https://doi.org/10.3390/s21155143>

[57] A. Baldazzi, L. Molinaro, J. Taborri, F. Margheritini, S. Rossi et al., Reliability of wearable sensors-based parameters for the assessment of knee stability. PLoS One **17**(9), e0274817 (2022). <https://doi.org/10.1371/journal.pone.0274817>

[58] K.S. Prakash, E. Andersen, V.C. von Einem, S.K. Muthalagu, P. Agarwal et al., Design and implementation of a wearable system based on a flexible capacitive sensor, monitoring knee laxity. Adv. Sens. Res. **2**(10), 2300058 (2023). <https://doi.org/10.1002/adsr.202300058>

[59] L. Xu, Q.K. Zhang, Z. Hu, C. Hua, L. Xue et al., Fully biodegradable piezoelectric nanogenerator based on cellulose/PLLA electrospun fibers with high-performance for mechanical energy harvesting. Colloids Surf. A Physicochem. Eng. Aspects **706**, 135813 (2025). <https://doi.org/10.1016/j.colsurfa.2024.135813>

[60] N. Yanar, T.Y.-S. Kim, J. Jung, D.K. Dinh, K. Choi et al., Boron nitride nanotube-aligned electrospun pvdf nanofiber-based composite films applicable to wearable piezoelectric sensors. ACS Appl. Nano Mater. **7**, 11715–11726 (2024). <https://doi.org/10.1021/acsanm.4c01296>

[61] K. Fu, P. Huang, S. Xu, L. Liu, X. Huang et al., Natural sucrose-assisted controllable porous PVDF-HFP films for self-powered tactile sensors with higher sensitivity. Surf. Interfaces **54**, 105252 (2024). <https://doi.org/10.1016/j.surfin.2024.105252>

[62] Y. Xue, Z. Weng, Q. Xiang, N. Liao, W. Xue, Magnetron sputtering preparation of flexible ZnO/AlN thin-films sensors with hybrid piezoelectric effect for broad-range human motions detection. Ceram. Int. **50**(23), 51429–51436 (2024). <https://doi.org/10.1016/j.ceramint.2024.10.059>

[63] Y. Yang, H. Pan, G. Xie, Y. Jiang, C. Chen et al., Flexible piezoelectric pressure sensor based on polydopamine-modified BaTiO_3_/PVDF composite film for human motion monitoring. Sens. Actuat. A Phys. **301**, 111789 (2020). <https://doi.org/10.1016/j.sna.2019.111789>

[64] S. Mirjalali, R. Bagherzadeh, S. Abrishami, M. Asadnia, S. Huang et al., Multilayered electrospun/electrosprayed polyvinylidene Fluoride+Zinc oxide nanofiber mats with enhanced piezoelectricity. Macromol. Mater. Eng. **308**(8), 2300009 (2023). <https://doi.org/10.1002/mame.202300009>

[65] K.-B. Kim, W. Jang, J.Y. Cho, S.B. Woo, D.H. Jeon et al., Transparent and flexible piezoelectric sensor for detecting human movement with a boron nitride nanosheet (BNNS). Nano Energy **54**, 91–98 (2018). <https://doi.org/10.1016/j.nanoen.2018.09.056>

[66] X. Hou, S. Zhang, J. Yu, M. Cui, J. He et al., Flexible piezoelectric nanofibers/polydimethylsiloxane-based pressure sensor for self-powered human motion monitoring. Energy Technol. **8**(3), 1901242 (2020). <https://doi.org/10.1002/ente.201901242>

[67] P. Snapp, C. Cho, D. Lee, M.F. Haque, S. Nam et al., Tunable piezoelectricity of multifunctional boron nitride nanotube/poly(dimethylsiloxane) stretchable composites. Adv. Mater. **32**(43), 2004607 (2020). <https://doi.org/10.1002/adma.202004607>

[68] J. Shim, D.I. Son, J.S. Lee, J. Lee, G.-H. Lim et al., BNNT-ZnO QDs nanocomposites for improving piezoelectric nanogenerator and piezoelectric properties of boron nitride nanotube. Nano Energy **93**, 106886 (2022). <https://doi.org/10.1016/j.nanoen.2021.106886>

[69] J. Zhang, S. Ye, H. Liu, X. Chen, X. Chen et al., 3D printed piezoelectric BNNTs nanocomposites with tunable interface and microarchitectures for self-powered conformal sensors. Nano Energy **77**, 105300 (2020). <https://doi.org/10.1016/j.nanoen.2020.105300>

[70] X. Sui, Q. Mu, J. Li, B. Zhao, H. Gu et al., High-performance flexible PLA/BTO-based pressure sensor for motion monitoring and human–computer interaction. Biosensors (Basel) **14**, 508 (2024). <https://doi.org/10.3390/bios14100508>
